# A systematic review and meta-analysis of the effectiveness of social norms messaging approaches for improving health behaviours in developed countries

**DOI:** 10.1038/s41562-025-02275-6

**Published:** 2025-09-22

**Authors:** Trisevgeni Papakonstantinou, Sarah Lynn Flecke, C. E. R. Edmunds, Rosina Cross, Anh Tran, Natalie Gold

**Affiliations:** 1 University College London, London, UK; 2 University of Innsbruck, Innsbruck, Austria; 3 UWE Bristol, Bristol, England; 4 University of Exeter, Exeter, England; 5 UK Health Security Agency, London, UK; 6 Kantar Public UK/London School of Economics, London, UK

**Keywords:** Human behaviour, Health sciences

## Abstract

Social norms approaches have been widely applied in health promotion as a cost-effective behaviour-change strategy, but have been little evaluated as a whole. We conducted a pre-registered systematic review and meta-analysis of randomized controlled trials using social norms messaging in developed countries targeted at changing health behaviours among 16+-year-olds to evaluate their effectiveness. Relevant studies were identified through searches in PsycINFO, Medline, Embase, Web of Science, TRIP, Cochrane and grey literature sources. Risk of bias was assessed independently by two reviewers using the Cochrane RoB 2 tool. A random-effects meta-analysis standardized effect sizes to Cohen’s *d*, assessed heterogeneity with *I*² and applied robust Bayesian meta-analysis to adjust for publication bias. Searches resulted in 89 studies (*n* = 85,759), which exhibited a small effect of social norms messaging on health behaviours (Cohen’s *d* = 0.1, 95% confidence interval (CI) [0.09, 0.19], *P* < 0.001). However, this effect disappeared after controlling for publication bias. We conducted moderator analyses, finding no significant differences from the overall effect for different types of social norms message, delivery modalities, health domains or target populations. The review is limited by the lack of studies assessing whether normative information changed participant perceptions, inconsistent use of manipulation checks, and high heterogeneity across studies in terms of target behaviour, population and intervention delivery, affecting the robustness of conclusions. Our analysis suggests that when appropriately controlling for publication bias, social norms messages are not effective at improving health behaviours. Thus, future attempts at improving public health should focus on alternative approaches.

## Abstract (web summary)

Social norms approaches are widely applied in health promotion. This pre-registered systematic review and meta-analysis of RCTs using social norms messaging in developed countries aimed to evaluate their effectiveness for changing health behaviours. An initial small effect disappeared when controlling for publication bias.

## Main

Communicating social norms has become increasingly popular over the past two decades as a simple and cost-effective approach to behaviour change^1,2^. Social norms are the informal or implicit rules that govern behaviour in particular social groups. According to an influential account in the behavioural literature^3^, they are underpinned by a preference for conforming with the behaviour of a reference group because individual group members expect others to do that too or because they think that others expect them to conform. This idea maps onto a distinction that is sometimes made between ‘descriptive norms’ (how others behave) and ‘injunctive norms’ (how others think we ought to behave). If people prefer to conform with their reference group, then giving information—a ‘social norms message’—about how members of the reference group behave or think people ought to behave should bring an individual’s behaviour in line with these norms. This is achieved without changing financial incentives, hence interventions that use social norm messages are sometimes considered a type of nudge^4^.

Social norms interventions also serve to challenge possible misperceptions people may have about what is normative. Applied initially in the context of college student substance abuse, social norms interventions focused on providing accurate information on peer attitudes and drinking behaviours to close the gap between the perceived and actual norm (see for example, ref. ^5^ for an early statement; taken up further by for example, refs. ^6–8^). Social norms interventions have since been applied to a range of different health behaviours: to promote positive changes to dietary behaviour^9,10^, improve the clinical behaviour of healthcare workers^11^, improve vaccination uptake^12^, increase contraceptive use^13^ and reduce prescription of antibiotics^14,15^. Such interventions encompass a variety of messaging, for instance:

- Social proof (stating what the reference group does): “Book your NHS health check. 6 million people have already attended”^16^.
- Social comparison (giving feedback on the recipient’s behaviour compared to the reference group): “The great majority (80%) of practices in England reduced or stabilized their antibiotic prescribing rates in 2016/17. However, your practice is in the minority that have increased their prescribing by more than 4%”^17^.
- Injunctive norms (stating what the reference group thinks people ought to do): “A lot of people aren’t aware that the typical student thinks their peers should eat five servings of fruits and vegetables each day. Students think you should eat more fruit and vegetables than you’d expect”^9^.

Although some reviews have found social norms messages to be an effective tool for behaviour change among healthcare practitioners (for example, refs. ^11,15^), other reviews have found limited or mixed results at changing health behaviours among the general population (for example, refs. ^10,18^). For effective use in health policy, it is crucial to understand which types of social norms message are (most) effective, as well as the relative effectiveness of different modes of delivery, and on which health behaviours and populations social norms messages are effective.

We conducted a systematic review and meta-analysis of randomized controlled trials that evaluate social norms message interventions across a range of health behaviours and delivery modalities. The pre-registered aim of this study is to determine what characteristics of social norms messages are most effective at changing health behaviours in people over the age of 16, including healthcare practitioners. Recognizing that there are cultural and structural differences in populations and healthcare systems, and working within the UK public health context, we were specifically looking for evidence of the effectiveness of social norms messaging on the general adult population and healthcare practitioners in developed countries (as defined by UNCTAD^19^).

The review seeks to answer the following research questions:

1. What type of social norm message is most effective at improving health behaviours (for example, social comparison, social proof, injunctive)?
2. What kind of normative comparison is most effective at improving health behaviours (for example, population-level comparison, comparison to average or typical individual, figure shown in absolute numbers or percentages)?
3. What modality of delivery is most effective at improving health behaviours (for example, physical letter, email or text message, poster, in-app or on-screen, verbal)?
4. For which health behaviours are social norms most effective at achieving positive change (for example, exercise, alcohol reduction, healthy eating, health screening, prescribing, vaccination)?
5. For which populations are social norms messages most effective in terms of changing health behaviours (for example, patients, health professionals)?

## Results

**Fig. 1:**
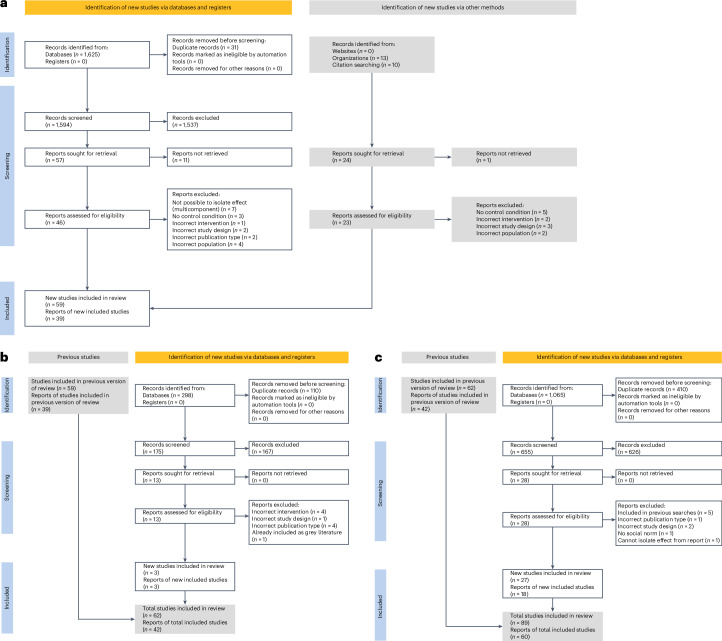
PRISMA flow diagrams. PRISMA flowcharts detailing the identification and screening of records for the systematic review and meta-analysis. **a**, Details for the search conducted in May 2021. **b**, Second search in June 2022. **c**, Final search conducted in April 2024.

A systematic review of the literature following the Preferred Reporting Items for Systematic Reviews and Meta-Analyses (PRISMA) guidelines^20^ found 110 papers. Of these papers, 60 had the information required for a meta-analysis. Some papers reported multiple studies, giving a total of 89 individual studies included in the analysis (see Table 1 for study characteristics). Figure 1 presents the PRISMA flow diagrams for the three searches.

**Table 1:** Description of included studies

Paper	Population	Type of social norms messaging	Mode of delivery	Domain of health behaviour	Follow-up point	Control	Intervention *N*	Control *N*	Cohen’s *d* (s.e.)
Anderson^74^	College students	Social proof - population statement - numeric data - proportion	In-app/on screen	Physical activity	1 week	Active	39	14	0.12 (0.31)
Anderson^74^	College students	Social proof - population statement - numeric data - proportion	In-app/on screen	Physical activity	2 weeks	Active	46	14	−0.13 (0.31)
Anderson^74^	College students	Social proof - population statement - numeric data - proportion	In-app/on screen	Physical activity	3 weeks	Active	30	13	0.01 (0.33)
Baretta^75^	Non-clinical general population	Combined: Injunctive + Social comparison - feedback comparing to average of population	In-app/on screen	Hand hygiene	32 days	Active	104	87	0.00 (0.15)
Beatty, 2018^76^	Non-clinical general population	Social comparison - feedback comparing to % or proportion population	Email/text message	Physical activity	10 weeks	No intervention	561	635	0.04 (0.06)
Bunten^27^	Non-clinical general population	Social proof - typical individual - no data	In-app/on screen	Diet	Immediate	Active	241	234	0.21 (0.09)
Chappell, 2021^77^	Healthcare professionals	Social comparison - feedback comparing to % or proportion population	Physical materials (letter, print)	Prescribing	5 months	No intervention	602	612	0.06 (0.06)
Choi^22^	Clinical patients	Social comparison - feedback comparing to % or proportion population	In-app/on screen	Mental health	Immediate	Active	255	273	−0.02 (0.09)
Clayton, 2021^78^	Non-clinical general population	Combined	Physical materials (letter, print)	Vaccination	6 months	No intervention	146	163	−0.09 (0.11)
Çoker, 2022^79^	Non-clinical general population	Social proof - population statement - no data	Poster/sign	Diet	Immediate	No intervention	22	22	0.01 (0.3)
Crane^45^	Clinical patients	Social comparison - feedback comparing to % or proportion population	In-app/on screen	Alcohol consumption	28 days	Active	98	81	0.04 (0.15)
Croker, 2009^80^	Non-clinical general population	Social comparison - feedback comparing to % or proportion population	Spoken word	Diet	Immediate	No intervention	284	256	0.14 (0.09)
Cunningham^52^	Non-clinical general population	Social comparison - feedback comparing to % or proportion population	Physical materials (letter, print)	Alcohol consumption	1 month	No intervention	435	206	−0.01 (0.08)
Cunningham, 2015^81^	Clinical patients	Social comparison - feedback comparing to % or proportion population	In-app/on screen	Alcohol consumption	3 months	No intervention	183	187	0.00 (0.1)
De Bauw^53^	Non-clinical general population	Social proof - population statement - numeric data	In-app/on screen	Diet	Immediate	Active	125	249	−0.07 (0.11)
De Bauw^53^	Non-clinical general population	Social proof - population statement - numeric data	In-app/on screen	Diet	Immediate	Active	125	249	0.07 (0.11)
Firkey^46^	College students	Social proof - population statement - numeric data - proportion	In-app/on screen	Sexual health	Immediate	Active	101	111	0.78 (0.14)
Galizzi^12^	Non-clinical general population	Social proof - population statement - numeric data - proportion	In-app/on screen	Vaccination	Immediate	Active	171	34	0.12 (0.19)
Galizzi^12^	Non-clinical general population	Social proof - population statement - numeric data - proportion	In-app/on screen	Vaccination	Immediate	Active	171	35	0.43 (0.19)
Galizzi^12^	Non-clinical general population	Social proof - population statement - numeric data - proportion	In-app/on screen	Vaccination	Immediate	Active	170	34	0.37 (0.19)
Galizzi^12^	Non-clinical general population	Social proof - population statement - numeric data - proportion	In-app/on screen	Vaccination	Immediate	Active	171	34	0.38 (0.19)
Galizzi^12^	Non-clinical general population	Social proof - population statement - numeric data - proportion	In-app/on screen	Vaccination	Immediate	Active	170	34	0.34 (0.19)
Gold^17^	Healthcare professionals	Social comparison with personalized feedback	Physical materials (letter, print)	Prescribing	1 year	No intervention	448	472	0.00 (0.08)
Gold^82^	Healthcare professionals	Social comparison - feedback comparing to % or proportion population	Physical materials (letter, print)	Prescribing	6 months	No intervention	324	302	0.00 (0.07)
Gorini^83^	Non-clinical general population	Social proof - population statement - numeric data - proportion	Physical materials (letter, print)	Screening	1 month	Active	1983	5566	0.18 (0.03)
Gorini^83^	Non-clinical general population	Social comparison - feedback comparing to % or proportion population	Physical materials (letter, print)	Screening	90 days	Active	1975	5566	0.11 (0.03)
Graupensperger^47^	Non-clinical general population	Social proof - population statement - numeric data - proportion	In-app/on screen	Alcohol consumption	1 month	Active	135	118	0.08 (0.13)
Gregorio-Pascual^21^	Non-clinical general population	Combined	Physical materials (letter, print)	Diet	2 weeks	Active	49	46	0.42 (0.21)
Gumussoy^84^	College students	Social proof - population statement - no data	Physical materials (letter, print)	Diet	Immediate	Active	30	30	0.57 (0.26)
Hallsworth, 2016^85^	Healthcare professionals	Social comparison - feedback comparing to % or proportion population	Physical materials (letter, print)	Prescribing	6 months	No intervention	791	790	0.19 (0.05)
Hansen^86,87^	Clinical patients	Social comparison - feedback comparing to average of population	In-app/on screen	Alcohol consumption	12 months	No intervention	365	358	−0.10, (0.07)
Havard^37^	Clinical patients	Social proof - population statement - numeric data - proportion	Physical materials (letter, print)	Alcohol consumption	6 weeks	No intervention	124	120	0.02 (0.13)
Huf^25^	Non-clinical general population	Social proof - population statement - numeric data - proportion	Email/text message	Screening	18 weeks	No intervention	1514	784	0.01 (0.04)
Huf^25^	Non-clinical general population	Social proof - population statement - numeric data	Email/text message	Screening	18 weeks	No intervention	1488	784	0.01 (0.04)
Koeneman, 2017^88^	Non-clinical general population	Social proof - population statement - no data	Physical materials (letter, print)	Physical activity	3 months	Active	10	9	0.71 (0.48)
Kroeze, 2008^89^	Non-clinical general population	Social comparison - feedback comparing to % or proportion population	Physical materials (letter, print)	Diet	6 months	Active	140	140	0.10 (0.12)
Lee^48^	College students	Social proof - population statement - numeric data - proportion	Not specified - ‘viewed’	Vaccination	Immediate	No intervention	428	109	0.33 (0.11)
Lewin, 2023^90^	Non-clinical general population	Social proof at population level - proportions	In-app/on screen	Alcohol consumption	Immediate	Active	84	84	0.26 (0.15)
Mahler^55^	College students	Social proof - population statement - numeric data - proportion	Spoken word	Sunscreen use	1 month	Active	22	8	−0.02 (0.41)
Mahler^55^	College students	Injunctive	Physical materials (letter, print)	Sunscreen use	1 month	Active	24	8	0.22 (0.41)
Mahler^55^	College students	Combined	Multimodal	Sunscreen use	1 month	Active	21	7	0.59 (0.44)
Marlow, 2021^91^	Non-clinical general population	Social proof - population statement - numeric data - proportion	Email/text message	Screening	Immediate	Active	270	226	0.11 (0.09)
Martens, 2015^92^	Non-clinical general population	Social comparison - feedback comparing to % or proportion population	Physical materials (letter, print)	Alcohol consumption	6 months	Active	163	162	0.07 (0.11)
Matkovic, 2021^93^	Non-clinical general population	Social proof - population statement - numeric data - proportion	In-app/on screen	Hand hygiene	Immediate	Active	68	65	−0.43 (0.18)
Michael, 2018^94^	Healthcare professionals	Social comparison - feedback comparing to % or proportion population	Multimodal	Prescribing	12 months	Active	51	58	0.11 (0.20)
Mollen^50^	Non-clinical general population (including college students)	Social proof at population level - no numbers	In-app/on screen	Diet	Immediate	Active	33	41	0.82 (0.33)
Mollen^50^	Non-clinical general population (including college students)	Social proof at individual level - numerical information	In-app/on screen	Diet	Immediate	Active	40	41	0.85 (0.32)
Mollen^50^	Non-clinical general population (including college students)	Injunctive	In-app/on screen	Diet	Immediate	Active	37	41	0.47 (0.33)
Montanaro, 2018^95^	College students	Social comparison - feedback comparing to % or proportion population	In-app/on screen	Sexual health	3 months	No intervention	31	28	−0.30 (0.26)
Nijssen, 2022^96^	Medical centre visitors	Social proof at population level - proportions	Audio recorded message	Smoking	Immediate	Active	1890	727	−0.05 (0.04)
Nix^51^	College students	Social comparison - feedback comparing to % or proportion population	Email/text message	Diet	1 week	Active	42	41	0.47 (0.22)
Perkins^43^	Non-clinical general population	Social proof - population statement - numeric data - proportion	Multimodal	Alcohol consumption	18 months (4 time points)	No intervention	218	162	0.99 (0.11)
Persell, 2016^97^	Healthcare professionals	Social comparison - feedback comparing to % or proportion population	Email/text message	Prescribing	1 year	Active	14	14	0.44 (0.38)
Priebe^57^	College students	Social proof - population statement - numeric data - proportion	Email/text message	Physical activity	15 days	Active	51	160	−0.07 (0.16)
Robinson^58^	College students	Social proof - typical individual - numeric data	Poster/sign	Diet	Immediate	Active	39	42	0.38 (0.22)
Robinson^9^	College students	Social proof - typical individual - numeric data	Multimodal	Diet	Immediate	Active	38	33	0.21 (0.24)
Robinson^9^	College students	Social proof - typical individual - numeric data	Multimodal	Diet	Immediate	Active	21	13	0.49 (0.36)
Robinson^9^	College students	Injunctive	Multimodal	Diet	Immediate	Active	22	14	−0.33 (0.34)
Sacarny, 2018^98^	Healthcare professionals	Social comparison - feedback comparing to % or proportion population	Physical materials (letter, print)	Prescribing	2 years	Active	2527	2528	0.34 (0.03)
Sallis, 2019a^59^	Non-clinical general population	Social comparison - feedback comparing to % or proportion population	Physical materials (letter, print)	Diet	5 months	Active	1270	1327	0.41 (0.04)
Sallis, 2019b^24^	Non-clinical general population	Social proof - population statement - no data	Physical materials (letter, print)	Screening	6 months	Active	800	204	0.06 (0.08)
Sallis, 2019b^24^	Non-clinical general population	Social proof - population statement - no data	Physical materials (letter, print)	Screening	6 months	Active	723	203	0.20 (0.08)
Sallis, 2019b^24^	Non-clinical general population	Social proof - population statement - no data	Physical materials (letter, print)	Screening	6 months	Active	754	203	0.19 (0.08)
Sallis, 2019b^24^	Non-clinical general population	Social proof - population statement - no data	Physical materials (letter, print)	Screening	6 months	Active	778	204	0.20 (0.08)
Schmidtke^99^	Healthcare professionals	Social proof - typical individual - numeric data	Physical materials (letter, print)	Vaccination	3 months	Active	1885	628	0.00 (0.05)
Schmidtke^99^	Healthcare professionals	Injunctive	Physical materials (letter, print)	Vaccination	3 months	Active	1885	629	0.00 (0.05)
Schmidtke^99^	Healthcare professionals	Combined	Physical materials (letter, print)	Vaccination	3 months	Active	1885	628	0.00 (0.05)
Siegel^60^	Non-clinical general population	Social proof - population statement - numeric data - proportion	In-app/on screen	Organ donation	Immediate	No intervention	235	212	0.32 (0.10)
Siegel^60^	Non-clinical general population	Social proof - population statement - numeric data - proportion	In-app/on screen	Organ donation	-Immediate	No intervention	246	259	0.30 (0.09)
Staudt^100^	Non-clinical general population	Social comparison with personalized feedback	Physical materials (letter, print)	Alcohol	3, 6 and 12 months	No intervention	815	831	0.03 (0.05)
Teo^101^	Veterans, non-clinical population	Social proof - population statement - no data	Physical materials (letter, print)	Appointment attendance	Immediate	Active	4353	4916	−0.01 (0.02)
Teo^101^	Veterans, non-clinical population	Social proof - population statement - no data	Physical materials (letter, print)	Appointment attendance	Immediate	Active	2250	1641	−0.07 (0.03)
Thomas^26^	College students	Social proof - typical individual - numeric data	Poster/sign	Diet	Immediate	Active	36	8	0.14 (0.39)
Thomas^26^	College students	Social proof - population statement - numeric data	Poster/sign	Diet	Immediate	Active	28	8	−0.09 (0.40)
Thomas^26^	College students	Social proof - typical individual - numeric data	Poster/sign	Diet	1 day	Active	37	8	0.58 (0.39)
Thomas^26^	College students	Social proof - population statement - numeric data	Poster/sign	Diet	1 day	Active	39	8	0.66 (0.39)
Thorndike, 2016^102^	Non-clinical general population	Social comparison - feedback comparing to average of population	Physical materials (letter, print)	Diet	3 months	No intervention	877	858	0.03 (0.05)
Torrente^23^	Healthcare professionals	Social comparison - feedback comparing to average of population	Email/text message	Prescribing	6 months	Active	906	905	0.10 (0.05)
Updegraff^61^	Non-clinical general population	Social proof - population statement - no data	Poster/sign	Hand hygiene	5 months	No intervention	14	15	1.19 (0.41)
van Bavel, 2014^103^	Non-clinical general population	Social comparison - feedback comparing to % or proportion population	In-app/on screen	Physical activity	Immediate	No intervention	400	400	0.05 (0.07)
Wagner^104^	Healthcare professionals	Social comparison - feedback comparing to % or proportion population	Email/text message	Prescribing	12 months	No intervention	207	217	0.16 (0.12)
Wagner^104^	Healthcare professionals	Social comparison - feedback comparing to % or proportion population	Email/text message	Prescribing	12 months	No intervention	216	217	0.20 (0.12)
Waite^105^	Non-clinical general population	Social proof - population statement - no data	In-app/on screen	Screening	Immediate	Active	91	89	−0.02 (0.15)
Waite^105^	Non-clinical general population	Social proof - population statement - no data	In-app/on screen	Screening	Immediate	Active	91	89	0.06 (0.15)
Wally^106^	College students	Combined	In-app/on screen	Physical activity	8 days	No intervention	43	17	0.53 (0.29)
Wally^106^	College students	Social comparison - feedback comparing to average of population	In-app/on screen	Physical activity	8 days	No intervention	43	17	0.62 (0.29)
Wilding^107^	Clinical patients	Social proof - population statement - numeric data	Physical materials (letter, print)	Screening	16 weeks	No intervention	3583	3590	−0.02 (0.02)
Young, 2013^108^	College students	Social proof - typical individual - no data	In-app/on screen	Sexual health	Immediate	No intervention	24	25	0.78 (0.09)
Young, 2013^108^	Non-clinical general population	Social proof - typical individual - no data	In-app/on screen	Sexual health	Immediate	Active	79	75	−0.35 (0.16)

### Types of intervention

Of the 89 studies included, 22 focused on diet, 12 on screening, 10 on vaccination, 10 on alcohol consumption, 10 on prescribing, 9 on physical activity and 4 on sexual health. Twelve studies were grouped as ‘other’: 3 studies on hand hygiene, 3 on sunscreen use, 2 on organ donation, 2 on appointment attendance, 1 on mental health and 1 on smoking. The 3 studies on sunscreen use were part of the same paper.

Most studies (*n* = 47) reported on interventions in the general population. Twenty-three studies focused specifically on college students, 13 on healthcare professionals and 6 on clinical patients. The studies included diverse delivery methods for social norms interventions: 29 used physical materials (letters or printed leaflets), 33 were delivered on-screen through mobile apps, websites, or similar displays, 10 were sent via email or text messages and 10 employed multiple modalities or audio, such as spoken word combined with images or text. In addition, 7 interventions delivered social norms messages through posters or signs.

Interventions included various types of both descriptive and injunctive social norms message. The majority of studies (*n* = 79) concentrated on descriptive social norms messaging, while 4 studies utilized injunctive social norms messaging (for example, “A lot of people aren’t aware that the typical student thinks their peers should eat five servings of fruits and vegetables each day. Students think you should eat more fruit and vegetables than you’d expect”^9^.), and 6 studies employed a combination of both descriptive and injunctive social norms messages (for example, “You thought that _____ % of college students try to avoid consuming sugar sweetened drinks. On average actually 90% of college students try to avoid consuming sugar-sweetened drinks”^21^.).

A further classification was made to distinguish between types of descriptive social norms message. There were 27 studies reporting on interventions using social comparison messaging with personalized feedback, 22 of which presented feedback comparing the individual to a percentage or proportion of the population (for example, “you have some difficulty bouncing back from stressful situations. Your resilience level is lower than 40% of males aged 18–29. Smiling Mind has some good tools to build your resilience”^22^), and 5 comparing the individual to the population average (for example, “you prescribe more than the average of PAMI doctors”^23^). There were 37 studies using social proof messaging without comparison or personalized feedback. Seven studies reported on interventions using social proof in the form of a population statement without numeric data (for example, “In Southwark, thousands of people like you have attended their health check and benefited from personalized health advice”^24^.). Six studies reported on interventions using social proof in the form of a population statement with absolute numeric data (for example, “Last year 12,000 women in Hillingdon took part in cervical screening. Your cervical smear test is due. To book please call <GP phone number>”^25^) and 23 had a statement with proportion numeric data (for example, “Last year in Hillingdon 7 out of 10 women took part in cervical screening. Your cervical smear test is due. To book please call <GP phone number>”^25^). Seven studies used interventions with social proof messaging in the form of a statement about the typical individual, presenting numerical data (for example, “Did you know most students eat a lot more vegetables than you might realize? Although, a lot of people aren’t aware, the typical student eats over three servings of vegetables each day”^26^), and three studies used a statement about the typical individual but without any numeric data (for example, “Swap to this product chosen by customers who buy similar groceries to you”^27^).

In a substantial portion of the studies (*n* = 34), outcomes were measured immediately after the intervention was delivered. In the remaining included studies (*n* = 55), follow-up periods varied from 1 day to 2 years, with the majority (*n* = 40) falling within the range of 1–6 months. Table 1 provides a detailed breakdown of follow-up times. Following the pre-registered analysis plan, when outcomes were assessed at multiple follow-up time points, we included the results from the furthest time point reported.

### Risk of bias

**Fig. 2:**
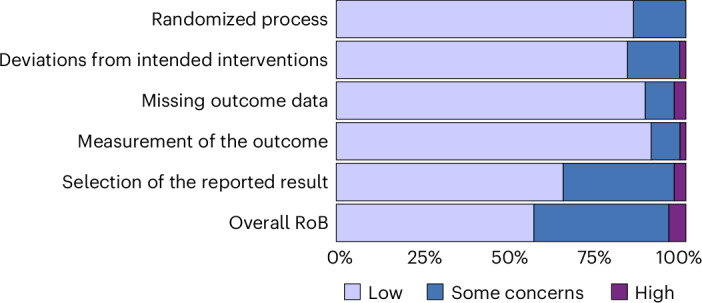
‘Risk of bias’ assessment summary. Breakdown of ‘risk of bias’ assessment.

A risk of bias assessment using the Cochrane RoB 2 tool^28^ showed that out of the 89 studies, most (*n* = 34) had low concerns, 23 had moderate concerns, and 3 were deemed to have a high risk. The primary concerns in most studies centred around two key aspects: bias resulting from deviations from the intended interventions and bias in the selection of the reported results. Figure 2 presents a breakdown of the ‘risk of bias’ assessment across all domains.

### Effect sizes

We observed heterogeneous results among the included studies, with 4 studies exhibiting large positive effects (Cohen’s *d* above 0.8), 9 studies with a moderate positive effect (Cohen’s *d* between 0.5 and 0.8), 22 studies with a small positive effect (Cohen’s *d* between 0.2 and 0.5) and 36 studies exhibiting effects below Cohen’s *d* 0.2. We also observed 18 studies displaying trivial to small negative effects (Cohen’s *d* between −0.01 and −0.4).

### Meta-analysis

**Fig. 3:**
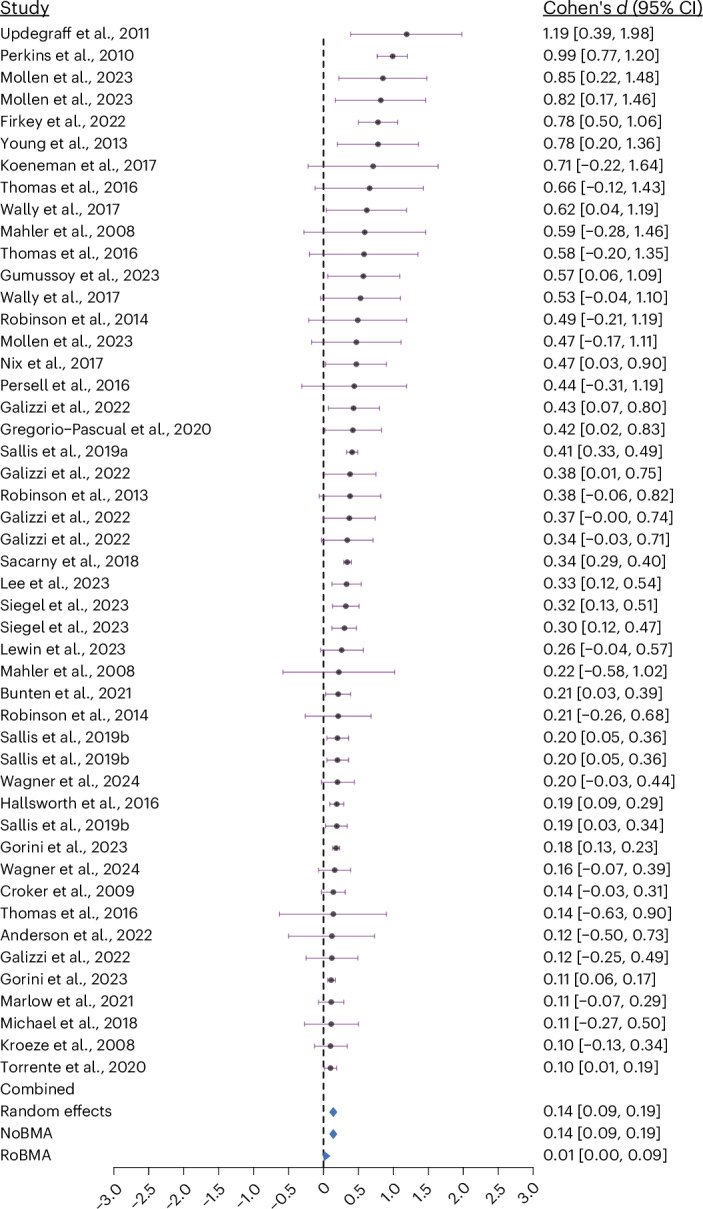
Forest plot of studies with effect sizes ≥0.10. Forest plot showing individual study effect sizes above Cohen’s *d* = 0.10 (*k* = 48) with corresponding 95% CIs. Each dot represents the estimated effect size from a single study, and the horizontal bars represent the 95% CI of that estimate. The diamond shapes at the bottom represent combined effect size estimates from random-effects meta-analysis, NoBMA and RoBMA models. Statistical tests conducted were two-sided. No corrections were made for multiple comparisons.

**Fig. 4:**
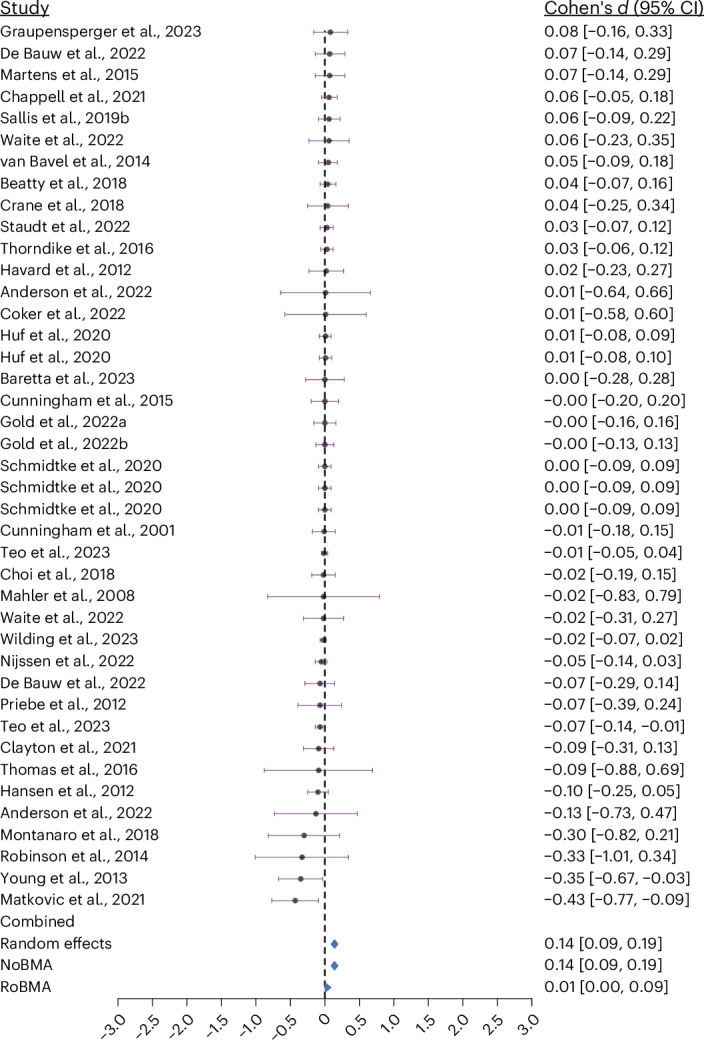
Forest plot of studies with effect sizes <0.10. Forest plot showing individual study effect sizes below Cohen’s *d* = 0.10 (*k* = 41) with corresponding 95% CIs. Each dot represents the estimated effect size from a single study, and the horizontal bars represent the 95% CI of that estimate. The diamond shapes at the bottom represent combined effect size estimates from random-effects meta-analysis, NoBMA and RoBMA models. Statistical tests conducted were two-sided. No corrections were made for multiple comparisons.

A linear random-effects model found a small statistically significant effect of social norms interventions on health behaviours (Cohen’s *d* = 0.14, 95% CI [0.09, 0.19], *P* < 0.001) (Figs. 3 and 4). This effect was reliable across pre-registered sensitivity analyses, with only small differences, including the removal of influential outliers (Cohen’s *d* = 0.11, 95% CI [0.08, 0.14], *P* < 0.001) and the removal of studies with overall ratings of ‘high’ and ‘some concerns’ in the ‘risk of bias’ assessment (Cohen’s *d* = 0.19, 95% CI [0.12, 0.26], *P* < 0.001). There is moderate variability in the effect sizes, as indicated by the estimated between-study heterogeneity of *τ* = 0.18 (95% CI [0.15, 0.25]). The predicted effect size ranged from *d* = −0.43 to 1.19, indicating that adverse effects cannot be ruled out. A power analysis based on the median number of participants for the intervention and control groups indicated that a Cohen’s *d* of 0.05 could be detected with 85% power, while a subgroup difference of 0.1 could be detected with 79% power.

### Moderator analyses

We examined four moderator variables: type of social norm messaging, mode of delivery, health behaviour and target population. Correlations among the four moderators were all large and significant. A multiple meta-regression model that included all variables as predictors yielded high variance inflation factor scores, indicating non-tolerable multicollinearity^29^. Due to this issue, we avoided using a multiple meta-regression approach, as it would probably produce unreliable results. Instead, we initially tested each moderator variable in separate simple meta-regression models, followed by Bayesian model-averaged meta-regression and robust Bayesian meta-regression models for each moderator.

Moderator analyses showed significant variation in effect sizes across different types of social norm message, modes of delivery, targeted behaviours and populations. The omnibus test for social norm type was significant (QM(9) = 38.76, *P* < 0.001, *τ* = 0.18), although no specific categories yielded significant deviations from the grand mean.

The delivery format also moderated effects significantly (QM(5) = 40.31, *P* < 0.001, *τ* = 0.17). Of the delivery modes, only physical materials (for example, leaflets, brochures) were associated with a significant, albeit small, negative deviation from the grand mean (Cohen’s *d* = −0.10, 95% CI [−0.22, 0.02], *P* = 0.034), even though the confidence interval spans zero. All other formats, including digital and audio/multimodal channels, did not significantly influence outcomes. Differences by target behaviour were also significant (QM(8) = 37.20, *P* < 0.001, *τ* = 0.18), but none of the behavioural domains examined showed significant deviations.

Finally, population subgroup analyses indicated significant moderation (QM(4) = 46.27, *P* < 0.001, *τ* = 0.16). Interventions delivered to university students were associated with a small but significant deviation from the grand mean (Cohen’s *d* = 0.17, 95% CI [0.04, 0.31], *P* < 0.001). No significant effects were observed among clinical patients, healthcare professionals or the general non-clinical population.

### Addressing publication bias

In light of recent findings that evidence for the effect of nudging interventions does not remain significant after adjusting for publication bias^30^, we re-evaluated our data with similar adjustments. Specifically, we employed robust Bayesian meta-analysis^31^ to account for potential publication bias.

**Fig. 5:**
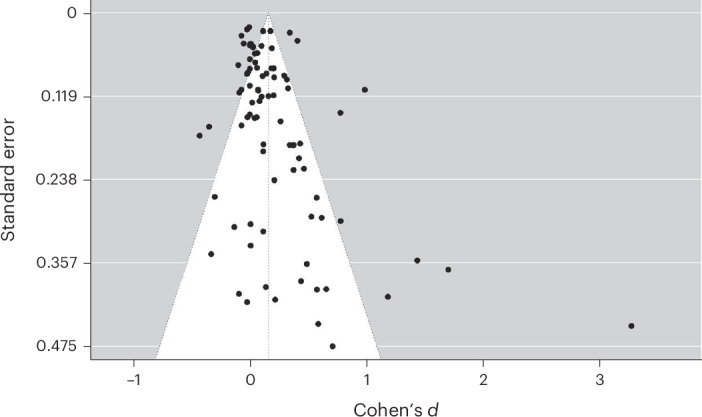
Funnel plot of study effect sizes. Funnel plot showing the distribution of standard errors of individual studies. Each observation is represented as a function of its effect size and standard error.

Visual inspection of a funnel plot presenting the relationship between effect sizes and their standard errors revealed an overrepresentation of positive effect sizes in studies with low precision (Fig. 5). Although an Egger’s test^32^ showed significant evidence for funnel plot asymmetry (*z* = 3.57, *P* < 0.001), it is important to note that such tests and visual inspection methods, including funnel plots, are often underpowered and insufficient for conclusively identifying publication bias. Simulation studies^33^ have demonstrated that more robust methods, such as Bayesian model-averaged meta-analytic approaches with publication bias adjustments, should be applied irrespective of visual or statistical indications of bias from funnel plots or Egger’s tests.

As a robustness check, we first tested our hypotheses using a Bayesian model-averaged meta-analytic model and then applied the same model with publication bias adjustment (RoBMA), which accounts for potential publication bias more reliably. Results are detailed in Table 1.

A Bayesian model-averaged meta-analytic model with weakly informative priors (*N*(0, 1) and *N*(0, 0.5)) showed the same overall effect (Cohen’s *d* = 0.14, 95% credible intervals (CrI) [0.09, 0.19], Bayes factor (BF)_01_ = 0.00). This effect was reliable across prior sensitivity analyses, with strongly informative priors (*N*(0.5, 0.1)) leading to a slightly larger but still small estimate (Cohen’s *d* = 0.16, 95% CrI [0.11, 0.21], BF_01_ = 0.00), and flat improper priors (*N*(0, 1,000)) showing the same effect (Cohen’s *d* = 0.14, 95% CrI [0.09, 0.19], BF_01_ = 0.00).

After adjusting for publication bias via robust Bayesian meta-analysis, the effect of social norms interventions on health behaviours was no longer statistically significant (Cohen’s *d* = 0.01, 95% CrI [0.00, 0.09]). The Bayes factor for this effect indicates strong evidence for the null hypothesis of no effect (BF_01_ = 0.11; see refs. ^34,35^) and there was very strong evidence for the presence of publication bias (BF_pb_ = 259.537). This model also showed substantial variability in the effect sizes, the estimated between-study heterogeneity being *τ* = 0.17 (95% CI [0.13, 0.21]).

**Fig. 6:**
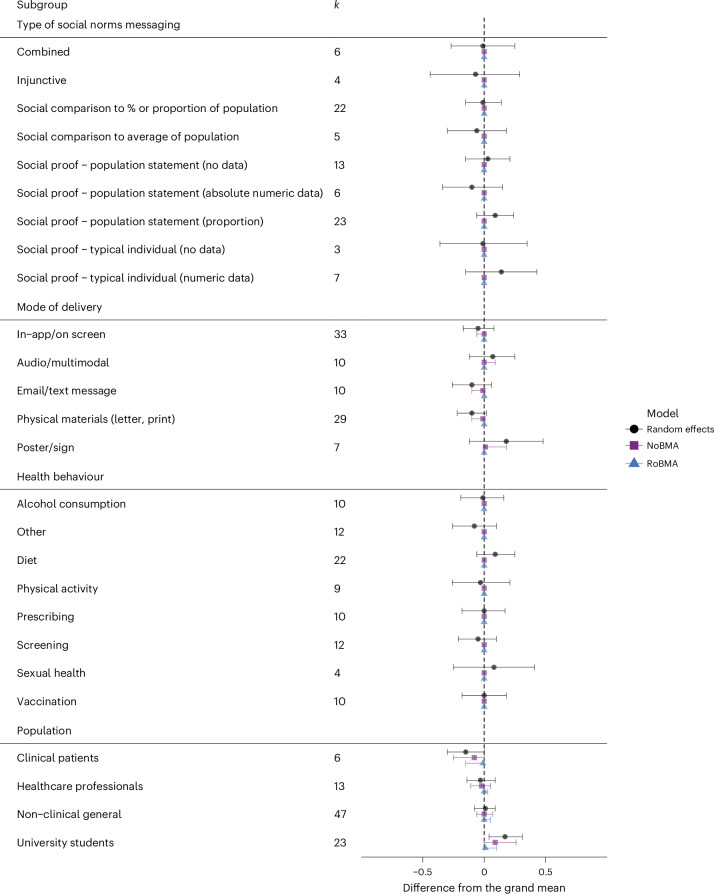
Moderation analysis of subgroups. Forest plot of moderation analyses showing differences from the grand mean for various subgroups of social norms messaging interventions, including results from the frequentist random-effects meta-regression, NoBMA and RoBMA models. Each point represents the estimated difference from the grand mean for that subgroup, and horizontal lines represent 95% CIs (for the frequentist model) and CrIs (for the Bayesian models) around those estimates. Estimates are derived from three models: random-effects (black circles), NoBMA (purple squares) and RoBMA (blue triangles). Data are presented as point estimates ± 95% CIs or CrIs. Statistical tests conducted were two-sided. No corrections were made for multiple comparisons.

We repeated the moderation tests, following the same procedure of applying a Bayesian unadjusted model and a Bayesian bias-adjusted model. For both Bayesian unadjusted and bias-adjusted models, none of the predictor levels showed significant differences from the overall effect for any of the moderator variables examined. Bayes factors for all moderator variables showed strong evidence in favour of the null hypothesis. Results are detailed in Table 2. Bayesian estimates based on weakly informative priors still show very small effects for the subgroup of messages presenting numeric data from a ‘typical individual’, messages delivered through posters or signs, the target domain of diet and the subpopulation of university students, although the credible intervals span zero (Table 2). The observed effects in the frequentist and unadjusted Bayesian model disappeared after adjusting for publication bias (see Fig. 6).

**Table 2:** Results of the main and moderator analyses using a frequentist random-effects model, NoBMA and RoBMA

Combined	*N*	Random effects	NoBMA	RoBMA
	89	0.14 [0.09, 0.14], *τ*(89) = 0.18	0.14 [0.09, 0.19], BF_01_ = 0.00	0.01 [0.00, 0.09], BF_01_ = 0.11
**Type of social norms messaging**				
Omnibus	89	QM(d.f. = 9) = 38.76, *P* < 0.001, *τ*(89) = 0.18	BF_01_ = 0.00	BF_01_ = 1,000
Combined	6	−0.01 [−0.27, 0.25], *P* = 0.916	0.00 [0.00, 0.00]	0.00 [0.00, 0.00]
Injunctive	4	−0.07 [−0.44, 0.29], *P* = 0.576	0.00 [0.00, 0.00]	0.00 [0.00, 0.00]
Social comparison to % or proportion of population	22	−0.01 [−0.15, 0.14], *P* = 0.897	0.00 [0.00, 0.00]	0.00 [0.00, 0.00]
Social comparison to average of population	5	−0.06 [−0.30, 0.18], *P* = 0.470	0.00 [0.00, 0.00]	0.00 [0.00, 0.00]
Social proof - population statement (no data)	13	0.03 [−0.15, 0.21], *P* = 0.649	0.00 [0.00, 0.00]	0.00 [0.00, 0.00]
Social proof - population statement (absolute data)	6	−0.10 [−0.34, 0.15], *P* = 0.263	0.00 [0.00, 0.00]	0.00 [0.00, 0.00]
Social proof - population statement (proportion)	23	0.09 [−0.06, 0.24], *P* = 0.096	0.00 [0.00, 0.00]	0.00 [0.00, 0.00]
Social proof - typical individual (no data)	3	−0.01 [−0.36, 0.35], *P* = 0.949	0.00 [0.00, 0.00]	0.00 [0.00, 0.00]
Social proof - typical individual (numeric data)	7	0.14 [−0.15, 0.43], *P* = 0.188	0.01 [0.00, 0.00]	0.00 [0.00, 0.00]
**Mode of delivery**				
Omnibus	89	QM(d.f. = 5) = 40.31, *P* < 0.001, *τ*(89) = 0.17	BF_01_ = 15.15	BF_01_ = 142.86
In-app/on screen	33	−0.05 [−0.17, 0.08], *P* = 0.322	0.00 [−0.06, 0.00]	0.00 [0.00, 0.00]
Audio/multimodal	10	0.07 [−0.12, 0.25], *P* = 0.346	0.00 [0.00, 0.09]	0.00 [0.00, 0.00]
Email/text message	10	−0.10 [−0.29, 0.06], *P* = 0.119	−0.01 [−0.10, 0.00]	0.00 [0.00, 0.00]
Physical materials	29	−0.10 [−0.22, 0.02], *P* = 0.034	−0.01 [−0.10, 0.00]	0.00 [0.00, 0.00]
Poster/sign	7	0.18 [−0.12, 0.48], *P* = 0.134	0.01 [0.00, 0.18]	0.00 [0.00, 0.00]
**Health behaviour**				
Omnibus	89	QM(d.f. = 8) = 37.20, *P* < 0.001, *τ*(89) = 0.18	BF_01_ = 1,000	BF_01_ = 2,522.54
Alcohol consumption	10	−0.01 [−0.19, 0.16], *P* = 0.825	0.00 [0.00, 0.00]	0.00 [0.00, 0.00]
Other	12	−0.08 [−0.26, 0.10], *P* = 0.208	0.00 [0.00, 0.00]	0.00 [0.00, 0.00]
Diet	22	0.09 [−0.06, 0.25], *P* = 0.102	0.01 [0.00, 0.00]	0.00 [0.00, 0.00]
Physical activity	9	−0.03 [−0.26, 0.21], *P* = 0.764	0.00 [0.00, 0.00]	0.00 [0.00, 0.00]
Prescribing	10	0.00 [−0.18, 0.17], *P* = 0.951	0.00 [0.00, 0.00]	0.00 [0.00, 0.00]
Screening	12	−0.05 [−0.21, 0.10], *P* = 0.364	0.00 [0.00, 0.00]	0.00 [0.00, 0.00]
Sexual health	4	0.08 [−0.25, 0.41], *P* = 0.497	0.00 [0.00, 0.00]	0.00 [0.00, 0.00]
Vaccination	10	0.00 [−0.18, 0.18], *P* = 0.991	0.00 [0.00, 0.00]	0.00 [0.00, 0.00]
**Population**				
Omnibus	89	QM(d.f. = 4) = 46.27, *P* < 0.001, *τ*(89) = 0.16	BF_01_ = 0.90	BF_01_ = 13.89
Clinical patients	6	−0.15 [−0.30, 0.00], *P* = 0.015	−0.08 [−0.25, 0.00]	−0.01 [−0.15, 0.00]
Healthcare professionals	13	−0.03 [−0.14, 0.09], *P* = 0.529	−0.02 [−0.11, 0.05]	0.00 [0.00, 0.04]
Non-clinical general	47	0.01 [−0.08, 0.09], *P* = 0.880	0.00 [−0.06, 0.07]	0.00 [0.00, 0.05]
University students	23	0.17 [0.04, 0.31], *P* = 0.002	0.09 [0.00, 0.26]	0.01 [0.00, 0.10]

The analyses were conducted using a random-effects model with the combined sample size (*N*) of 89 unless otherwise specified. For each comparison, 95% CrIs are provided. In the case of the NoBMA and RoBMA models, Bayes factor (BF_01_) is reported. The statistical tests used include omnibus tests for group differences, with the corresponding QM statistic and degrees of freedom (d.f.). Where applicable, *P* values are reported. No corrections for multiple comparisons were applied. The direction of effects (positive or negative) and the corresponding credible intervals are displayed for each model. The *P* values are indicated as exact values when available.

## Discussion

The results of our meta-analysis, which included 89 studies in 60 papers, indicated that social norms interventions have a small but statistically significant positive effect on health behaviours. This effect, however, no longer holds after adjusting for publication bias. A funnel plot revealed an overrepresentation of positive effect sizes in studies with low power, indicating publication bias. After adjusting for publication bias through robust Bayesian meta-analysis, we found no effect and moderate evidence for the absence of an effect. We found substantial variability in the effect sizes of individual studies. Overall, our analysis showed that while the omnibus tests indicated significant moderation for the pre-registered variables (type of messaging, mode of delivery, health domain and population), none of the individual levels significantly differed from the grand mean for any variable tested after adjusting for publication bias, suggesting no meaningful moderation effect at the level of specific subgroups.

Our findings contradict much of the previous literature that has found social norms messaging interventions to be effective at improving health behaviours^9,11,15^. It must be noted, however, that the effects observed in the literature are consistently very small, and results in many cases are mixed (for example, refs. ^10,18^). To the best of our knowledge, all previous meta-analyses of social norms interventions on health behaviours were conducted using frequentist analytic methods. The results of our frequentist models are largely consistent with previous literature, showing small to null effects. A key contribution this study makes to the literature is the replication of these analyses using a more robust analytic approach that accounts for conservative priors and publication bias.

Our finding that there is no effect of social norms messaging after adjusting for publication bias is consistent with the wider literature on the effectiveness of nudging. Reference ^30^ found that evidence for the effectiveness of nudging interventions from a meta-analysis in ref. ^35^ no longer held after adjusting for publication bias. This relates to the main issue identified through the ‘risk of bias’ assessment of the included studies, which was the selection of the reported result (Fig. 2). A potential explanation for the discrepancy between our conclusions and those of previous reviews, as further evidenced by the difference between the unadjusted and bias-adjusted results, is that positive results are more likely to be selected and published. This is in line with more recent findings from ref. ^36^ which found that despite pre-registration, many randomized controlled trials conducted by nudge units provide insufficient detail in their pre-analysis plans and final reports, making it difficult to assess the extent of selective reporting. Notably, focusing the analysis on papers with low risk of bias yielded a higher overall effect estimate for the frequentist model, warranting further investigation. Given this phenomenon, an interesting avenue for future research could be to conduct meta-analyses focusing solely on papers published after the replication crisis publicity and the advent of the open science movement.

In our meta-analysis, we only extracted data about primary hypotheses and favoured intention-to-treat over per-protocol analyses. Therefore, significant results in per-protocol or subgroup analyses may not have been included. It is possible that social norm interventions are more effective in particular subgroups (for example, risky drinkers^37^). However, we considered our approach to be a reasonable one for pooling data in this domain. Studies are rarely powered for subgroup analysis, which increases the chance of false positives in those tests, and nudges are often rolled out across whole populations, with a claim that small average effects are impactful over a large population (for example, refs. ^38,39^). Focusing on population averages addresses the literature on its own terms.

Omnibus tests of moderation were significant for all variables; however, when examining individual-variable levels against the overall effect, we found that none differed from the grand mean significantly. All four moderator models still showed substantial heterogeneity with *τ* ranging from 0.20 to 0.24, suggesting that heterogeneity even among moderator levels may contribute to the discrepancies in effect sizes and yield results that are less meaningful^40,41^. This heterogeneity could explain why our findings differ from those reported in previous studies, although the evidence from previous literature is mixed^41^.

The type of social norm messaging did not moderate the effectiveness of social norms interventions on health behaviours. Our findings contradict two previous meta-analyses, one showing that descriptive norms can affect intentions to undertake positive healthcare behaviours^42^, and another finding that interventions that used social comparison had a positive, albeit very small, effect on the clinical behaviour of healthcare workers with a standardized mean difference (SMD) of 0.06 (95% CI [0.04–0.08])^11^. In both cases, we found that our frequentist models replicated the results, with the effective types of message all being descriptive norms and social comparisons with a small effect. However, these effects no longer hold in the RoBMA model.

Mode of delivery did not moderate the effect of social norms interventions on health behaviours. It should be noted that the studies in each category, of which all but one came from the same paper^26^, were fairly heterogeneous (except those on posters and signs). Nevertheless, our findings are reasonably consistent with those of ref. ^11^; results of the frequentist model on delivery by email, in writing and in mixed format were all consistent with the average effect size they found (which was very small). Once more, when adjusting for publication bias through RoBMA, the effects seem to no longer hold. One study in this analysis stands out due to the much larger effect with a very precise estimate^43^. Unlike many other studies included in this analysis, which provide a one-time message or single feedback letter, ref. ^43^ employed an intensive media intervention on a statewide level over the course of 15 months, which is the longest intervention period of any of the included studies.

The effect size of social norm messaging interventions did not vary across different health domains. Diet was the domain with the highest estimated difference from the overall effect, although this difference was not significant. The direction of the effect is consistent with other reviews that have found that conveying normative information—including not only social norms messaging, but also modelling of behaviour and implicit messages conveyed by portion size—is effective in changing dietary behaviour^9,10^. We did not find a statistically significant effect of feedback in the domain of prescribing, which contrasts with ref. ^11^ that found a small effect of social norm messages on prescribing behaviours (SMD 0.11, 95% CI [0.09–0.13], *n* = 21), and ref. ^15^ that found an effect of social norms feedback on antibiotic prescribing. The latter included a different sample of papers, including lower-quality designs (non-randomized studies with concurrent controls and controlled before-and-after studies, as well as randomized controlled trials (RCTs)). Both these meta-analyses did not include some more recent studies that found null effects (for example, ref. ^17^).

Among all subgroups, only college students showed a significant effectiveness of social norms interventions according to the frequentist model, but this effect disappeared after adjusting for publication bias. To the best of our knowledge, there have been no other meta-analyses of social norms interventions on the health behaviours of college students. We did not find a statistically significant effect of social norms interventions on behaviours of healthcare workers. This contrasts with ref. ^11^, which found a very small effect of social norms messages on healthcare workers’ clinical behaviour (95% CI [0.07–0.10]). It is worth noting that, while most studies included sample populations with unknown previous levels of the target behaviour, some studies included sample populations due to their above- or below-average levels of target behaviour (that is, higher-risk groups), such as low mental resilience^22^ and excessive levels of alcohol consumption^44^. It is possible that normative messages affect higher-risk groups differently. Future research could investigate this further.

Social norms messaging is supposed to work via a preference for conformity with the behaviour of the group. It is motivated by the key assumption that perceptions of peers’ attitudes and behaviour is incorrect. Social norms messages aim to correct recipients’ perceptions of social norms and therefore positively influence their behaviour. Of the 89 studies included in this review, only 10 elicited participants’ perceived norms pre-intervention^12,21,26,45–51^. Thus, it remains unclear whether the normative information changed participant perceptions of the norm at all. Moreover, only 17 studies included manipulation checks, verifying participants’ understanding and awareness of the norm^21,26,45–48,50,52–61^. Many of the studies we covered were field experiments, so it is difficult to measure anything other than behaviour. Nevertheless, future studies could consider whether it is possible to elicit people’s perceptions of norms, both before and after receiving the message, which may shed some light on the mixed effectiveness of messaging.

There was moderate heterogeneity in the results of the studies in our meta-analysis (*τ* = 0.22), with some studies exhibiting moderate to large positive effects and a few exhibiting negative effects. This may not be surprising given that the included studies are themselves heterogeneous in terms of target behaviour, population and mode of delivery. We remain cautious in interpreting meta-analytic averages when the studies exhibit high heterogeneity^40,41^. It may be more useful to look for explanations of the heterogeneity^42^. Indeed, the pre-registered aim of our study was to conduct moderator analyses, to give guidance on what characteristics of social norm messages are more effective at achieving positive health behaviour change, and on what types of behaviour and population they are more effective on. Nevertheless, we did not find any significant effects among our selected moderators.

Social norms interventions appear to have limited effectiveness in improving health behaviours. Our meta-analysis suggests that any small effects previously reported could be the result of selective reporting. The presence of moderate heterogeneity in all our models indicates that we should interpret the meta-analytic average with caution. The pooling of effect sizes from substantially different intervention types, populations and outcome targets means that we cannot draw firm conclusions from the pooled effect alone. However, the examination of pre-registered moderators suggests that this type of heterogeneity is not the reason why results might be inconsistent; moderators did not account for any considerable percentage of the heterogeneity and yielded null results when comparing individual subgroups to the overall pooled effect. Heterogeneity may be stemming from other factors, such as mode of measurement or follow-up time^62^. Key questions for further research are the longevity of any effects of social norms messaging and the effectiveness of repeatedly using the same intervention. At a policy level, future interventions for improving public health outcomes should explore alternative approaches.

## Methods

The meta-analysis methods adhered to the Cochrane guidelines for conducting systematic reviews of interventions^63^ and followed the PRISMA standards^20^. The protocol for this study was registered on PROSPERO (https://www.crd.york.ac.uk/prospero/display_record.php?ID=CRD42021253063) before conducting data extraction.

### Inclusion criteria

1. Study type: randomized controlled trial
2. Language: reported in English
3. Population: people aged 16 or older in developed countries (see the United Nations Conference on Trade and Development 2023 country classifications^19^ for further details)
4. Intervention:
  - Social norms message interventions targeting single health behaviours alone or multiple health behaviours, or healthcare workers’ behaviours.
  - Includes messages that provide normative information about the behaviour of others within a relevant reference group, which may include social comparisons (target performance relative to a reference group), social proof (information about what the reference group does), injunctive norms (information about what ought to be done) or a combination thereof.
  - Messages can be delivered through any modality, and they can include pictures, as well as text or verbal messages.
  - It must be possible to assess the effectiveness of the social norms aspect of the intervention; thus, if there is a multicomponent intervention, then the control condition must allow the effectiveness of the social norm component to be assessed, or the social norms element must be the main active ingredient of the intervention. All types of control were considered, including studies with both active and passive controls.
5. Outcomes: the study should include objective behavioural outcomes or intention to perform behavioural outcomes. Examples of relevant outcomes: alcohol consumption, exercise or physical activity, fruit and vegetable consumption, prescribing, handwashing, uptake of screening, uptake of health checks and uptake of vaccination.

### Exclusion criteria

Studies were excluded if:

1. They were conference abstracts, unpublished theses, discussion papers, editorials, policy articles, and epidemiological, cross-sectional or longitudinal observational studies, or non-randomized controlled trials.
2. They did not include a social norms approach to health behaviour change.
3. Social norms interventions did not target a health behaviour.
4. It was not possible to isolate the effect of social norms, for example, a multicomponent intervention with a no-intervention control where the social norms component was not the main active ingredient.
5. They targeted the following populations or health behaviours: defecation, female genital mutilation (FGM), alcohol or cannabis consumption in college students or school students, populations in developing countries.
6. The population whose behaviour was targeted included adolescents or children under the age of 16.

Our exclusion criteria were designed to exclude literature on interventions that are not applicable to the healthcare context of developed countries and do not target the general adult population (of patients or health care practitioners). To achieve this, we excluded literature on the basis of two factors: location (populations in developing countries) and health behaviours that are not relevant to majority of the general population in developed countries (such as defecation and FGM). We also excluded literature pertaining to interventions on alcohol or cannabis consumption in college students or school students. These interventions target a problem specific to a very particular type of community and population (young educated adults, generally all living together). Further, there is an extensive body of literature on this population, which would have disproportionately influenced our results (see for example, ref. ^64^ for a review).

### Search strategy

A combination of focused searches were undertaken, including searches of bibliographic databases and searches for grey literature.

Searches were conducted on the following databases:

- PsycINFO
- Medline
- Embase
- Psychology and Behavioral Science Collection
- Web of Science
- TRIP
- Cochrane

We also searched for grey literature by asking for studies from providers on the Cabinet Office Behavioural Insights procurement framework, and behavioural science research and consultancy groups, including the Behavioural Insights Team (BIT), Penn SoNG (University of Pennsylvania), CogCo (the Cognition Company) and Behaviour Change for Good (Wharton), and by searching PROSPERO for other registered reviews that might be relevant and asking their authors for reference lists.

### Search terms and limits

Search terms were derived from the inclusion criteria in relation to study design (that is, RCT), population (adults), intervention (social norms) and outcomes (health behaviours). We used both free text and thesaural terms. They were combined using Boolean and proximity operators to search the electronic databases stated above^63^ as follows (also see Supplementary Table 1):

[social norms OR ((social OR descriptive OR injunctive OR subjective) AND (norm OR norms)) OR (peer OR family OR social) AND influence*]

AND

[letter OR text messaging OR (feedback OR leaflet* OR campaign* OR program* or letter* OR text* OR email OR e-mail OR (change* behavio?r*))]

AND

(drug prescriptions OR prescrib* OR antimicrobial stewardship OR ((antimicrobial OR anti-microbial) stewardship))

OR

(infection control* OR (hand* AND (wash* OR hygiene)))

OR

(overweight OR obesity OR (overweight OR obes*) OR (weight AND (control OR gain OR loss)))

OR

(exercise OR sedentary behavio?r OR (physical activity OR exercis* OR ‘resistance training’))

OR

(diet OR feedback behaviour OR (snack* OR diet* OR nutrition) OR ‘eating behavio?r’ OR ((fruit OR vegetable*) AND (consum* OR eat*)))

OR

(sexual behaviour OR unsafe sex) OR (sexual AND (health OR behavio?r)) OR (‘condom use’ OR ‘using condom*’ OR ‘safe sex’)

OR

(oral health OR ((oral OR dental) AND (health OR hygiene OR care)))

OR

(substance-related disorders OR (‘drug use’ OR ‘using drugs’ OR ‘drug abuse’ OR ‘drug misuse’ OR ‘drug mis-use’) OR (drug* AND tak*))

OR

((alcoholic intoxication OR alcoholism OR binge drinking) OR (‘binge drinking’ OR ‘alcohol consum*’ OR ‘alcohol misuse’ OR ‘alcohol mis-use’))

OR

(screen time OR (screen viewing OR ‘screen time’))

OR

(public health OR ‘public health’)

AND

[RCT OR ‘randomi?ed controlled trial*’]

Several limits were placed on the search, including a language filter to select only studies published in English, and an RCT filter to select only RCTs, since we anticipated a large number of studies and wanted to restrict to the gold standard for provision of evidence of effectiveness. The specific search terms used were not pre-registered in the protocol.

Exploratory searches were conducted in February 2021, and primary searches were conducted in March 2021 and re-run in April 2022 to guarantee that newly published studies are included in the systematic review. Our cut-off date for retrieval of papers and grey literature for the first search was 31 May 2021, and 17 June 2022 for the updated search. A final database search was conducted in March 2024.

### Study screening

Rayyan QCRI reference management software^65^ was used to manage records. Once a record of the number of references downloaded from each database was recorded, they were combined, with duplicate references removed.

Initially, titles and abstracts were screened by two reviewers (out of N.G., T.P., C.E.R.E., S.L.F. and a research assistant). If the studies did not meet the inclusion criteria (or met the exclusion criteria) they were excluded. Where titles met the inclusion criteria (but did not meet the exclusion criteria), or it was unclear, the citation was retained and the full text of the paper obtained. Disagreements were resolved by discussion. Full texts were screened by two reviewers.

### Data extraction

Data were extracted by one reviewer, and a second reviewer checked for correctness and completeness of extracted data. There was one extractor and one checker per paper. Reviewers and checkers were taken from a pool including T.P., C.E.R.E. S.L.F., a research assistant and R.C.

Raw data on social norms intervention methods were extracted from each study using a data extraction template. Reviewers tested the data extraction template using three randomly selected articles. Raw descriptive data from each study were extracted by one reviewer and checked by a second reviewer to ensure consistency of social norms coding. Statistical data from included studies, including the type of outcome data, the statistical significance testing conducted and measures of effect sizes used, were extracted and compiled in a raw data table (see Supplementary Table 1). Where the necessary value for the analysis was neither available nor reported in the study, authors were contacted directly and asked to provide any missing data, where applicable.

We extracted data regarding results of the main hypothesis (or hypotheses) only, not from additional subgroup analyses or exploratory analyses. When authors provided both intention-to-treat (ITT) and per-protocol (PP) results, we extracted the more conservative ITT values.

### ‘Risk of bias’ (quality) assessment

The quality of the papers included was assessed for risk of bias using the Cochrane RoB 2 tool^28^. Risk of bias was assessed independently by two reviewers.

### Analytic approach

Analyses were conducted in R (v.4.4.1)^66^ using the packages metafor^67^ (v.4.6-0) and RoBMA^68^ (v.3.1). Effects of included studies were not uniformly reported (for example, some studies included means and standard deviations, others odds ratios, yet others included regression coefficients and so on). To adequately compare the studies, results needed to be converted to a common effect size, Cohen’s *d*.

Following this standardization, we performed a random-effects regression^69^, employing the restricted maximum likelihood estimation method, to obtain an overall effect. We displayed the results of individual studies and syntheses visually using forest plots. Heterogeneity of the effect sizes were calculated using the *I*^2^ statistic^70^, and the variance of the underlying distribution of true effect sizes was estimated with the *τ* statistic^63,71,72^. We also performed the analysis following the removal of studies with overall ratings of ‘high’ and ‘some concerns’ in the ‘risk of bias’ assessment^65^.

We conducted moderator analyses to determine the effectiveness that specific characteristics of interventions have on intervention outcomes. The moderators were type of social norm messaging, mode of delivery, health behaviour, target population and quality of studies as determined by the ‘risk of bias’ assessment. We conducted a comparison of each group with the grand mean of all groups for each moderator. We further interrogated the significant moderators by including other variables in the model as a robustness check.

The moderators were based on the pre-registered protocol. The categorization of types of social norms message was derived inductively from the search results (that is, looking at the text of the social norm messages we found) to group similar messages so that they could be compared in the meta-analysis. Each social norm message was classified by one of our pool of reviewers and the coding checked by a second reviewer. The categorization was pre-registered before the data analysis began.

Finally, we tested for publication bias and applied robust Bayesian meta-analysis, a bias correction technique that makes multimodel inferences about the presence or absence of an effect, heterogeneity and publication bias, by applying selection models that estimate relative publication probabilities and modelling the relationship between effect sizes and standard errors^68^. As a robustness check, we first tested our main hypotheses using a Bayesian model-averaged meta-analytic model. We then applied the same model with an adjustment for publication bias (RoBMA). We repeated the same process for each of the four moderation models. We conducted prior sensitivity analyses for all the models.

These analyses were pre-registered on the Open Science Framework at

https://osf.io/jkd4v/?view_only=c509214920af454684762a76b940209f.

### Reporting summary

Further information on research design is available in the Nature Portfolio Reporting Summary linked to this article.

## Supplementary information

Supplementary Information Search Terms, and Supplementary Tables 1 and 2.

Reporting Summary

## Data Availability

The data for this paper’s analyses were compiled by the authors from the studies identified in the systematic review. Searches were conducted on the following databases: PsycINFO (https://www.apa.org/pubs/databases/psycinfo), Medine (https://www.nlm.nih.gov/medline/medline_home.html), Embase (https://www.elsevier.com/en-gb/products/embase), Psychology and Behavioral Science Collection (https://www.ebsco.com/products/research-databases/psychology-behavioural-sciences-collection), Web of Science (https://clarivate.com/academia-government/scientific-and-academic-research/research-discovery-and-referencing/web-of-science/), TRIP (https://www.tripdatabase.com/) and Cochrane (https://www.cochrane.org/). Data used in the analysis are available on the Open Science Framework repository at 10.17605/OSF.IO/JKD4V (ref. ^73^).

## Code availability

The code to replicate the analysis is available on the Open Science Framework repository at 10.17605/OSF.IO/JKD4V (ref. ^73^).
