# Supplementary material for: A systematic review and meta-analysis of the effectiveness of social norms messaging approaches for improving health behaviours in developed countries
Source: Nat Hum Behav. 2025 Sep 22;9(12):2632–50. doi: 10.1038/s41562-025-02275-6 (PMC12727529; doi:10.1038/s41562-025-02275-6)
Supplement: Supplementary file 1 — Search Terms, and Supplementary Tables 1 and 2. [file 41562_2025_2275_MOESM1_ESM.pdf]

nature human behaviour

Supplementary information https://doi.org/10.1038/s41562-025-02275-6

A systematic review and meta-analysis of the effectiveness of social norms messaging approaches for improving health behaviours in developed countries

In the format provided by the authors and unedited

Medline Search Terms

Database(s): Ovid MEDLINE(R) ALL 1946 to July 09, 2021
Search Strategy:

#	Searches
1	*Social Norms/
2	((social or descriptive or injunctive or subjective) adj2 (norm or norms)).tw,kw.
3	((peer* or family or social) adj2 influence*).tw,kw.
4	normative.tw,kw.
5	1 or 2 or 3 or 4
6	*Letter/
7	*Text messaging/
8	(feedback or leaflet* or campaign* or program* or letter* or text* or email or e-mail or (change* adj1 behavio?r*)).tw,kw.
9	6 or 7 or 8
10	*Drug Prescriptions/
11	prescrib*.tw,kw.
12	Antimicrobial Stewardship/
13	((antimicrobial or anti-microbial) adj1 stewardship).tw,kw.
14	10 or 11 or 12 or 13
15	(infection adj2 control*).tw,kw.
16	(hand* adj1 (wash* or hygiene)).tw,kw.
17	15 or 16
18	*Overweight/

19	*Obesity/
20	(overweight or obes*).tw,kw.
21	(weight adj2 (control or gain or loss)).tw,kw.
22	18 or 19 or 20 or 21
23	*Exercise/
24	"sedentary behavio?r".tw,kw.
25	(physical activity or exercis* or "resistance training").tw,kw.
26	23 or 24 or 25
27	exp *Diet/
28	*Feeding Behavior/
29	(snack* or diet* or nutrition).tw,kw.
30	"eating behavio?r*".tw,kw.
31	((fruit or vegetable*) adj1 (consum* or eat*)).tw,kw.
32	27 or 28 or 29 or 30 or 31
33	*sexual behavior/ or *unsafe sex/
34	(sexual adj1 (health or behavio?r)).tw,kw.
35	("condom use" or "using condom*" or "safe sex").tw,kw.
36	33 or 34 or 35
37	*Oral Health/
38	((oral or dental) adj1 (health or hygiene or care)).tw,kw.
39	37 or 38
40	exp *Substance-Related Disorders/

41	("drug use" or "using drugs" or "drug abuse" or "drug misuse" or "drug mis-use").tw,kw.
42	(drug* adj1 tak*).tw,kw.
43	40 or 41 or 42
44	*alcoholic intoxication/ or *alcoholism/ or *binge drinking/
45	("binge drinking" or "alcohol consum*" or "alcohol misuse" or "alcohol mis-use").tw,kw.
46	44 or 45
47	*screen time/
48	(screen viewing or "screen time").tw,kw.
49	47 or 48
50	exp *Public Health/
51	"public health".tw,kw.
52	50 or 51
53	14 or 17 or 22 or 26 or 32 or 36 or 39 or 43 or 46 or 49 or 52
54	5 and 9 and 53
55	(RCT or " randomi?ed controlled trial*").ti.
56	54 and 55
57	limit 54 to randomized controlled trial
58	5 and 53
59	55 and 58
60	limit 58 to randomized controlled trial

Table S1.

Complete description of included studies with original reported statistics

Paper	Country	Domain of health behaviour	Population	Type of social norms messaging	Mode of delivery	Follow-up point	Intervention duration	Cohen's d (SE)	Intervention N	Intervention mean	Intervention SD	Intervention SE	Intervention uptake proportion	Intervention CI lower bound	Intervention CI upper bound	Control N	Control mean	Control SD	Control SE	Control uptake proportion	Control CI lower bound	Control CI upper bound	Mean difference	Odds ratio	CI lower bound	CI upper bound	Regression coefficient	Regression coefficient SE
Anderson, 2022	Canada	Physical Activity	University students	Social proof - population statement - numeric data - proportion	In-app / on screen	1 week		0.12, (0.31)	39	5.83	1.21					14	5.69	1.22										
Anderson, 2022	Canada	Physical Activity	University students	Social proof - population statement - numeric data - proportion	In-app / on screen	2 week		-0.13, (0.31)	46	5.53	1.21					14	5.69	1.22										
Anderson, 2022	Canada	Physical Activity	University students	Social proof - population statement - numeric data - proportion	In-app / on screen	3 week		0.01, (0.33)	30	5.70	1.23					13	5.69	1.22										
Baretta, 2023	Switzerland	Hand Hygiene	Non-clinical general population	Combined	In-app / on screen	32 days	32 days	0, (0.15)	104							87												
Beatty, 2018	US	Physical Activity	Non-clinical general population	Social comparison - feedback comparing to % or proportion population	Email / text message	10 weeks	Weekly email - 10 weeks	0.04, (0.06)	561							635											-0.03	0.08
Bunten, 2021	UK	Diet	Non-clinical general population	Social proof - typical individual - no data	In-app / on screen	-		0.21, (0.09)	241							234											0.34	0.25
Chappell, 2021	New Zealand	Prescribing	Healthcare professionals	Social comparison - feedback comparing to % or proportion population	Physical materials (letter, print)	5 months	Single letter	0.06, (0.06)	602				0.16			612				0.18				0.89				

Choi, 2018	Australia, New Zealand, the United States, or the United Kingdom	Mental health	Clinical patients	Social comparison - feedback comparing to % or proportion population	In-app / on screen	-	Message in app	-0.02, (0.09)	255				0.45			273				0.47				0.96				
Clayton, 2021	USA	Vaccination	Non-clinical general population	Combined	Physical materials (letter, print)	6 months	-	-0.09, (0.11)	146							163											0.00	0.00
Coker, 2022	UK	Diet	Non-clinical general population	Social proof - population statement - no data	Poster / sign	-	2 weeks	0.01, (0.3)	22							22											7e-04	0.03
Crane, 2018	UK	Alcohol consumption	Clinical patients	Social comparison - feedback comparing to % or proportion population	In-app / on screen	28 days	Embedded in modules	0.04, (0.15)	98	-4.10	14.93					81	-3.52	12.87										
Croker, 2009	UK	Diet	Non-clinical general population	Social comparison - feedback comparing to % or proportion population	Audio/Multimodal	-	The satement was framed as a question as part of the interview	0.14, (0.09)	284	3.94	1.52			3.68	4.20	256	3.31	1.47			3.05	3.58						
Cunningham, 2001	Canada	Alcohol consumption	Non-clinical general population	Social comparison - feedback comparing to % or proportion population	Physical materials (letter, print)	1 month	Single pamphlet	-0.01, (0.08)	435	1.46	0.95					206	1.45	0.93										
Cunningham, 2015	Canada	Alcohol consumption	Clinical patients	Social comparison - feedback comparing to % or proportion population	In-app / on screen	3 months	Single report	0, (0.1)	183	20.80	0.72					187	20.80	0.70										
De Bauw, 2022	Belgium	Diet	Non-clinical general population	Social proof - population statement - numeric data	In-app / on screen	-	Single exercise	-0.07, (0.11)	125	0.18	1.65			0.47	-0.11	249	0.04	2.05			0.30	-0.21						

De Bauw, 2022	Belgium	Diet	Non-clinical general population	Social proof - population statement - numeric data	In-app / on screen	-	Single exercise	0.07, (0.11)	125	-0.19	1.71			0.11	-0.49	249	-0.11	0.63			0.12	-0.04						
Firkey, 2022	USA	Sexual Health	University students	Social proof - population statement - numeric data - proportion	In-app / on screen	-	-	0.78, (0.14)	101	7.44	2.53					111	5.20	3.16										
Galizzi, 2022	UK	Vaccination	Non-clinical general population	Social proof - population statement - numeric data - proportion	In-app / on screen	-	Single online message (mean duration 8.5 min)	0.12, (0.19)	171							34											3.03	3.67
Galizzi, 2022	UK	Vaccination	Non-clinical general population	Social proof - population statement - numeric data - proportion	In-app / on screen	-	Single online message (mean duration 8.5 min)	0.43, (0.19)	171							35											10.18	3.35
Galizzi, 2022	UK	Vaccination	Non-clinical general population	Social proof - population statement - numeric data - proportion	In-app / on screen	-	Single online message (mean duration 8.5 min)	0.37, (0.19)	170							34											9.80	3.79
Galizzi, 2022	UK	Vaccination	Non-clinical general population	Social proof - population statement - numeric data - proportion	In-app / on screen	-	Single online message (mean duration 8.5 min)	0.38, (0.19)	171							34											10.07	3.79
Galizzi, 2022	UK	Vaccination	Non-clinical general population	Social proof - population statement - numeric data - proportion	In-app / on screen	-	Single online message (mean duration 8.5 min)	0.34, (0.19)	170							34											9.10	3.80
Gold, 2022	UK	Prescribing	Healthcare professionals	Social comparison - feedback comparing to % or proportion population	Physical materials (letter, print)	6 months	-	0, (0.08)	324	0.00	1e-04					302	0.00	1e-04										
Gold, 2022b	UK	Prescribing	Healthcare professionals	Social comparison - feedback comparing to % or proportion population	Physical materials (letter, print)	1 year (intervention monthly over a year, have reported the average over the whole period)	Single letter	0, (0.07)	448	0.06	0.01					472	0.06	0.02									-0.01	-0.50

Gorini, 2023	Italy	Screening	Non-clinical general population	Social proof - population statement - numeric data - proportion	Physical materials (letter, print)			0.18, (0.03)	1983							5566								1.38	1.13	1.68		
Gorini, 2023	Italy	Screening	Non-clinical general population	Social comparison - feedback comparing to % or proportion population	Physical materials (letter, print)	90 days		0.11, (0.03)	1975							5566								1.23	1.00	1.50		
Graupensperger, 2023	USA	Alcohol Consumption	Non-clinical general population	Social proof - population statement - numeric data - proportion	In-app / on screen	1 month	1 month	0.08, (0.13)	135	2.90	5.20					118	3.40	6.80										
Gregorio-Pascual, 2020	US	Diet	Non-clinical general population	Combined	Physical materials (letter, print)	2 weeks	Single pamphlet	0.42, (0.21)	49	12.39	14.63	2.09				46	18.58	14.58	2.15									
Gumussoy, 2023	UK	Diet	University students	Social proof - population statement - no data	Physical materials (letter, print)	-	Single-exposure text	0.57, (0.26)	30	9.10	11.04			5.20	13.10	30	2.80	10.90			-1.10	6.70						
Hallsworth, 2016	UK	Prescribing	Healthcare professionals	Social comparison - feedback comparing to % or proportion population	Physical materials (letter, print)	6 months	Single letter	0.19, (0.05)	791							790												
Hansen, 2012	Denmark	Alcohol consumption	Clinical patients	Social comparison - feedback comparing to average of population	In-app / on screen	12 months	Single message	-0.1, (0.07)	365	27.70	15.60					358	26.40	9.90					-1.40		-3.40	0.60		
Havard, 2012	Australia	Alcohol consumption	Clinical patients	Social proof - population statement - numeric data - proportion	Physical materials (letter, print)	6 weeks	Single letter	0.02, (0.13)	124	-3.76	19.20					120		28.58										
Huf, 2020	UK	Screening	Non-clinical general population	Social proof - population statement - numeric data - proportion	Email / text message	18 weeks	Single message	0.01, (0.04)	1514							784								1.01	0.87	1.18		
Huf, 2020	UK	Screening	Non-clinical general population	Social proof - population statement - numeric data	Email / text message	18 weeks	Single message	0.01, (0.04)	1488							784								1.02	0.88	1.18		

Koenema n, 2017	Netherla nds	Physical Activity	Non-clinical general population	Social proof - population statement - no data	Physical materials (letter, print)	3 months	-	0.71, (0.48)	10				0.80			9				0.22				3.64				
Kroeze, 2008	Netherla nds	Diet	Non-clinical general population	Social comparison - feedback comparing to % or proportion population	Physical materials (letter, print)	6 months	Single letter	0.1, (0.12)	140	78.70	27. 73					140	81. 70	30 .7 6										
Lee 2023	US	Vaccinatio n	University students	Social proof - population statement - numeric data - proportion	Audio/Multi modal	-	Single exposure	0.33, (0.11)	428	0.11	0.9 3					109	- 0.2 0	0. 98										
Lewin, 2023	UK	Alcohol Consumpti on	Non-clinical general population	Social proof - population statement - numeric data - proportion	In-app / on screen	-	Single- exposure text	0.26, (0.15)	84	4.68	0.9 7					84	4.9 4	1. 02										
Mahler, 2008	USA	Sunscreen Use	University students	Social proof - population statement - numeric data - proportion	Audio/Multi modal	1 month	-	-0.02, (0.41)	22	-0.03	0.4 2					8	- 0.0 2	0. 47										
Mahler, 2008	USA	Sunscreen Use	University students	Injunctive	Physical materials (letter, print)	1 month	-	0.22, (0.41)	24	0.10	0.5 7					8	- 0.0 2	0. 47										
Mahler, 2008	USA	Sunscreen Use	University students	Combined	Audio/Multi modal	1 month	-	0.59, (0.44)	21	0.23	0.4 1					7	- 0.0 2	0. 47										
Marlow, 2021	GB	Screening	Non-clinical general population	Social proof - population statement - numeric data - proportion	Email / text message	-	-	0.11, (0.09)	270							226												
Martens, 2015	US	Alcohol Consumpti on	Non-clinical general population	Social comparison - feedback comparing to % or proportion population	Physical materials (letter, print)	6 months	10 minutes	0.07, (0.11)	163	8.20	10. 83					162	9.0 8	13 .1 8										
Matkovic, 2021	USA	Hand Hygiene	Non-clinical general population	Social proof - population statement - numeric data - proportion	In-app / on screen	-	-	-0.43, (0.18)	68	4.46	0.5 3					65	4.6 6	0. 39										

Michael, 2018	US	Prescribing	Healthcare professionals	Social comparison - feedback comparing to % or proportion population	Audio/Multimodal	12 months	NI	0.11, (0.2)	51							58							-0.80		-1.50	-0.10		
Mollen, 2023	Netherlands	Diet	Non-clinical general population	Social proof - population statement - no data	In-app / on screen	-	Single exposure to text	1.44, (0.35)	33	5.15	0.20					14	4.87	0.18										
Mollen, 2023	Netherlands	Diet	Non-clinical general population	Social proof - typical individual - numeric data	In-app / on screen	-	Single exposure to text	3.28, (0.45)	40	5.46	0.18					14	4.87	0.18										
Mollen, 2023	Netherlands	Diet	Non-clinical general population	Injunctive	In-app / on screen	-	Single exposure to text	1.71, (0.37)	37	5.19	0.19					13	4.87	0.18										
Montanaro, 2018	USA	Sexual Health	University students	Social comparison - feedback comparing to % or proportion population	In-app / on screen	3 months	Single exposure	-0.3, (0.26)	31	7.68	4.64					28	9.54	7.51										
Nijssen, 2022	Germany	Smoking	Non-clinical general population	Social proof - population statement - numeric data - proportion	Audio/Multimodal	-	Audio recording played every 5 minutes	-0.05, (0.04)	1890				0.44			727				0.40				1.10				
Nix, 2017	USA	Diet	University students	Social comparison - feedback comparing to % or proportion population	Email / text message	1 week	-	0.47, (0.22)	42	2.30	2.10					41	1.50	1.20										
Perkins, 2010	USA	Alcohol Consumption	Non-clinical general population	Social proof - population statement - numeric data - proportion	Audio/Multimodal	18 months (4 time points)	15 months (Jan 2002- March 2003)	0.99, (0.11)	218				0.02			162				0.12				0.17				
Persell, 2016	USA	Prescribing	Healthcare professionals	Social comparison - feedback comparing to % or proportion population	Email / text message	1 year	monthly email	0.44, (0.38)	14							14								0.45	0.18	1.11		
Priebe, 2012	Canada	Physical Activity	University students	Social proof - population statement - numeric data - proportion	Email / text message	15 days	15 days	-0.07, (0.16)	51	1.86	20.40					160	3.59	25.20										

Robinson, 2013	UK	Diet	University students	Social proof - typical individual - numeric data	Poster / sign	-	minutes	0.38, (0.22)	39	30.20	20.90					42	41.80	37.70										
Robinson, 2014	UK	Diet	University students	Social proof - typical individual - numeric data	Audio/Multimodal	-	-	0.21, (0.24)	38	57.61	47.01					33	47.55	47.81										
Robinson, 2014	UK	Diet	University students	Social proof - typical individual - numeric data	Audio/Multimodal	-	-	0.49, (0.36)	21	75.30	56.78					13	47.05	58.82										
Robinson, 2014	UK	Diet	University students	Injunctive	Audio/Multimodal	-	-	-0.33, (0.34)	22	32.27	32.80					14	47.05	58.82										
Sacarny, 2018	USA	Prescribing	Healthcare professionals	Social comparison - feedback comparing to % or proportion population	Physical materials (letter, print)	2 years	-	0.34, (0.03)	2527							2528							-1157.00		-1343.00	-970.00		
Sallis, 2019a	UK	Weight Management	Non-clinical general population	Social comparison - feedback comparing to % or proportion population	Physical materials (letter, print)	5 months	-	0.41, (0.04)	1270							1327								2.10	1.28	3.46		
Sallis, 2019b	UK	Screening	Non-clinical general population	Social proof - population statement - no data	Physical materials (letter, print)	6 months	-	0.06, (0.08)	800							204								1.12	0.87	1.43		
Sallis, 2019b	UK	Screening	Non-clinical general population	Social proof - population statement - no data	Physical materials (letter, print)	6 months	1 week	0.2, (0.08)	723							203								1.45	1.13	1.85		
Sallis, 2019b	UK	Screening	Non-clinical general population	Social proof - population statement - no data	Physical materials (letter, print)	6 months	1 week	0.19, (0.08)	754							203								1.40	1.10	1.79		
Sallis, 2019b	UK	Screening	Non-clinical general population	Social proof - population statement - no data	Physical materials (letter, print)	6 months	2 weeks	0.2, (0.08)	778							204								1.45	1.14	1.85		
Schmidtke, 2020	UK	Vaccination	Healthcare professionals	Social proof - typical individual - numeric data	Physical materials (letter, print)	3 months	3 months (letters mailed once)	0, (0.05)	1885		0.55		0.43	0.41	0.46	628		0.26		0.43	0.41	0.45		1.00				
Schmidtke, 2020	UK	Vaccination	Healthcare professionals	Injunctive	Physical materials (letter, print)	3 months	3 months (letters mailed once)	0, (0.05)	1885		0.44		0.43	0.41	0.45	629		0.26		0.43	0.41	0.45		1.00				

Schmidtke, 2020	UK	Vaccination	Healthcare professionals	Combined	Physical materials (letter, print)	3 months	3 months (letters mailed once)	0, (0.05)	1885		0.44		0.43	0.41	0.45	628		0.26		0.43	0.41	0.45		1.00				
Siegel, 2023	US	Organ donation	Non-clinical general population	Social proof - population statement - numeric data - proportion	In-app / on screen	-	Single exposure to text	0.32, (0.1)	235	51.28	30.29					212	41.83	28.93										
Siegel, 2023	US	Organ donation	Non-clinical general population	Social proof - population statement - numeric data - proportion	In-app / on screen	-	Single exposure to text	0.3, (0.09)	246	48.13	29.18					259	39.52	29.12										
Staudt, 2022	Germany	Alcohol Consumption	Non-clinical general population	Social comparison - feedback comparing to average of population	Physical materials (letter, print)	3, 6, and 12 months	Letter (3, at baseline, month 3 and month 6)	0.03, (0.05)	815	2.54	4.95					831	2.67	4.16										
Teo, 2023	USA	Appointment Attendance	Non-clinical general population	Social proof - population statement - no data	Physical materials (letter, print)		Single letter	-0.01, (0.02)	4353							4916								1.01	0.75	1.37		
Teo, 2023	USA	Appointment Attendance	Non-clinical general population	Social proof - population statement - no data	Physical materials (letter, print)		Single letter	-0.07, (0.03)	2250							1641								1.14	0.82	1.60		
Thomas, 2016	UK	Diet	University students	Social proof - typical individual - numeric data	Poster / sign	-	-	0.14, (0.39)	36	114.28	33.48					8	109.35	45.07										
Thomas, 2016	UK	Diet	University students	Social proof - population statement - numeric data	Poster / sign	-	-	-0.09, (0.4)	28	106.76	21.28					8	109.35	45.07										
Thomas, 2016	UK	Diet	University students	Social proof - typical individual - numeric data	Poster / sign	1 day	24 hrs	0.58, (0.39)	37	117.30	26.01					8	102.19	27.10										
Thomas, 2016	UK	Diet	University students	Social proof - population statement - numeric data	Poster / sign	1 day	24 hrs	0.66, (0.39)	39	117.01	21.59					8	102.19	27.10										
Thorndike, 2016	USA	Diet	Non-clinical general population	Social comparison - feedback comparing to average of population	Physical materials (letter, print)	3 months	3 months (letters mailed monthly)	0.03, (0.05)	877				0.52			858				0.49				1.05				

Torrente, 2020	Argentina	Prescribing	Healthcare professionals	Social comparison - feedback comparing to average of population	Email / text message	6 months	6 months	0.1, (0.05)	906							905							-0.40		-0.77	-0.04		
Updegraff, 2011	USA	Hand Hygiene	Non-clinical general population	Social proof - population statement - no data	Poster / sign	5 months	5 months	1.19, (0.41)	14							15											0.37	0.13
Wagner, 2024	USA	Prescribing	Healthcare professionals	Social comparison - feedback comparing to % or proportion population	Email / text message	12 months	-	0.16, (0.12)	207				0.28			108				0.37				0.75				
Wagner, 2024	USA	Prescribing	Healthcare professionals	Social comparison - feedback comparing to % or proportion population	Email / text message	12 months	-	0.2, (0.12)	216				0.25			109				0.37				0.69				
Waite, 2022	UK	Screening	Non-clinical general population	Social proof - population statement - no data	In-app / on screen	-	Single exposure	-0.02, (0.15)	91	5.63	0.50	0.05				89	5.64	0.50	0.05									
Waite, 2022	UK	Screening	Non-clinical general population	Social proof - population statement - no data	In-app / on screen	-	Single exposure	0.06, (0.15)	91	5.67	0.50	0.05				89	5.64	0.50	0.05									
Wally, 2017	USA	Physical Activity	University students	Combined	In-app / on screen	8 days	8 days	0.53, (0.29)	43							17							0.12		-0.01	0.25		
Wally, 2017	USA	Physical Activity	University students	Social comparison - feedback comparing to average of population	In-app / on screen	8 days	8 days	0.62, (0.29)	43							17							0.14		0.01	0.27		
Wilding, 2023	UK	Screening	Clinical patients	Social proof - population statement - numeric data	Physical materials (letter, print)	16 weeks	Single exposure	-0.02, (0.02)	3583				0.39			3590				0.41				0.96				
Young, 2013	USA	Sexual Health	University students	Social proof - typical individual - no data	In-app / on screen	-	-	0.78, (0.3)	24	4.94	0.24					25	4.48	0.87							-0.88	-0.05		
Young, 2013	mTurk	Sexual Health	Non-clinical general population	Social proof - typical individual - no data	In-app / on screen	-	-	-0.35, (0.16)	79	3.40	2.00					75	4.10	2.00							-1.40	-0.10		

van Bavel, 2014	Bulgaria, Croatia and Romania	Physical Activity	Non-clinical general population	Social comparison - feedback comparing to % or proportion population	In-app / on screen	-	-	0.05, (0.07)	400							400												

Table S2. Risk of Bias Assessment

Paper	Overall RoB	Randomization process	Deviations from intended interventions	Missing outcome data	Measurement of the outcome	Selection of the reported result
Anderson, 2022	Some concerns	Some concerns	Low	Some concerns	Low	High
Baretta, 2023	Some concerns	Low	Low	Low	Low	Some concerns
Beatty, 2018	High	Some concerns	Low	Low	Low	High
Bunten, 2021	Low	Low	Low	Low	Low	Low
Chappell, 2021	Low	Low	Low	Low	Low	Low
Choi, 2018	Low	Low	Low	Low	Low	Low
Clayton, 2021	Some concerns	Low	Low	Low	Low	Some concerns
Çoker, 2022	Low	Low	Low	Low	Low	Low
Crane, 2018	Some concerns	Low	Low	Some concerns	Low	Low
Croker, 2009	Low	Low	Low	Low	Low	Low
Cunningham, 2001	Low	Low	Low	Low	Low	Low

Cunningham, 2015	Low	Low	Low	Low	Low	Low
De Bauw, 2022	Some concerns	Some concerns	Low	Low	Low	Some concerns
Firkey, 2022	Some concerns	Low	Low	Low	Low	Some concerns
Galizzi, 2022	Some concerns	Some concerns	Some concerns	Low	Low	Some concerns
Gold, 2022a	Low	Low	Low	Low	Low	Low
Gold, 2022b	Low	Low	Low	Low	Low	Low
Gorini, 2023	Some concerns	Some concerns	Low	Low	Some concerns	Low
Graupensperger, 2023	Some concerns	Low	Low	Low	Low	Some concerns
Gregorio-Pascual, 2020	Low	Low	Low	Low	Low	Low
Gumussoy, 2023	Low	Low	Low	Some concerns	Low	Low
Hallsworth, 2016	Some concerns	Low	Some concerns	Low	Some concerns	Low

Hansen, 2012	High	Low	Low	High	High	Some concerns
Havard, 2012	Some concerns	Low	Some concerns	Some concerns	Low	Some concerns
Huf, 2020	Some concerns	Low	Some concerns	Low	Low	Low
Koeneman, 2017	High	Low	High	High	Some concerns	Some concerns
Kroeze, 2008	Low	Low	Low	Low	Low	Some concerns
Lee, 2023	Low	Some concerns	Low	Low	Low	Low
Lewin, 2023	Low	Low	Low	Low	Low	Low
Mahler, 2008	Low	Low	Low	Low	Low	Some concerns
Marlow, 2021	Low	Low	Low	Low	Low	Low
Martens, 2015	Some concerns	Low	Some concerns	Low	Low	Some concerns
Matkovic, 2021	Some concerns	Low	Some concerns	Low	Low	Low

Michael, 2018	Some concerns	Low	Low	Low	Low	Some concerns
Mollen, 2023	Low	Low	Low	Low	Low	Low
Montanaro, 2018	Low	Low	Low	Low	Low	Low
Nijssen, 2022	Some concerns	Some concerns	Some concerns	Low	Some concerns	Some concerns
Nix, 2017	Low	Low	Some concerns	Low	Low	Low
Perkins, 2010	Low	Low	Low	Low	Low	Low
Persell, 2016	Low	Low	Low	Low	Low	Low
Priebe, 2012	Some concerns	Low	Some concerns	Low	Low	Some concerns
Robinson, 2013	Low	Low	Low	Low	Low	Low
Robinson, 2014	Some concerns	Some concerns	Low	Low	Low	Some concerns
Sacarny, 2018	Low	Low	Low	Low	Low	Low
Sallis, 2019a	Low	Low	Low	Low	Low	Low
Sallis, 2019b	Low	Low	Low	Low	Low	Low
Schmidtke, 2020	Low	Low	Low	Low	Low	Low
Siegel, 2023	Low	Low	Low	Low	Low	Low

Staudt, 2022	Low	Low	Low	Low	Low	Low
Teo, 2023	Some concerns	Some concerns	Low	Low	Low	Some concerns
Thomas, 2016	Some concerns	Low	Low	Low	Low	Some concerns
Thorndike, 2016	Low	Low	Low	Low	Low	Low
Torrente, 2020	Low	Low	Low	Low	Low	Low
Updegraff, 2011	Some concerns	Low	Low	Low	Some concerns	Low
van Bavel, 2014	Some concerns	Low	Low	Some concerns	Low	Low
Wally, 2017	Low	Low	Low	Low	Low	Low
Wagner, 2024	Low	Low	Low	Low	Low	Low
Waite, 2022	Low	Low	Low	Low	Low	Low
Wilding, 2023	Low	Low	Low	Low	Low	Low
Young, 2013	Some concerns	Low	Low	Low	Low	Some concerns
