# Supplementary material for: A systematic review and meta-analysis of the effectiveness of social norms messaging approaches for improving health behaviours in developed countries
Source: Nat Hum Behav. 2025 Sep 22;9(12):2632–50. doi: 10.1038/s41562-025-02275-6 (PMC12727529; doi:10.1038/s41562-025-02275-6)
Supplement: Supplementary file 2 — Reporting Summary [file 41562_2025_2275_MOESM2_ESM.pdf]

nature portfolio

Corresponding author(s): Trisevgeni Papakonstantinou

Last updated by author(s): May 4, 2025

Reporting Summary

Nature Portfolio wishes to improve the reproducibility of the work that we publish. This form provides structure for consistency and transparency in reporting. For further information on Nature Portfolio policies, see our Editorial Policies and the Editorial Policy Checklist.

Statistics

For all statistical analyses, confirm that the following items are present in the figure legend, table legend, main text, or Methods section.

n/a	Confirmed	
☐	☒	The exact sample size (*n*) for each experimental group/condition, given as a discrete number and unit of measurement
☐	☒	A statement on whether measurements were taken from distinct samples or whether the same sample was measured repeatedly
☐	☒	The statistical test(s) used AND whether they are one- or two-sided<br>*Only common tests should be described solely by name; describe more complex techniques in the Methods section.*
☐	☒	A description of all covariates tested
☐	☒	A description of any assumptions or corrections, such as tests of normality and adjustment for multiple comparisons
☐	☒	A full description of the statistical parameters including central tendency (e.g. means) or other basic estimates (e.g. regression coefficient) AND variation (e.g. standard deviation) or associated estimates of uncertainty (e.g. confidence intervals)
☐	☒	For null hypothesis testing, the test statistic (e.g. *F*, *t*, *r*) with confidence intervals, effect sizes, degrees of freedom and *P* value noted<br>*Give P values as exact values whenever suitable.*
☐	☒	For Bayesian analysis, information on the choice of priors and Markov chain Monte Carlo settings
☐	☒	For hierarchical and complex designs, identification of the appropriate level for tests and full reporting of outcomes
☐	☒	Estimates of effect sizes (e.g. Cohen's *d*, Pearson's *r*), indicating how they were calculated

Our web collection on statistics for biologists contains articles on many of the points above.

Software and code

Policy information about availability of computer code

Data collection	Rayyan QCRI reference management software (no version applicable - accessed in 2021, 2022, 2024) , PRISMA 2020, Cochrane RoB 2 tool
Data analysis	Analyses were conducted using R (version 4.4.1) using the packages metafor (version 4.6-0) and RoBMA(version 3.1). Original analysis scripts are available at https://osf.io/jkd4v/

For manuscripts utilizing custom algorithms or software that are central to the research but not yet described in published literature, software must be made available to editors and reviewers. We strongly encourage code deposition in a community repository (e.g. GitHub). See the Nature Portfolio guidelines for submitting code & software for further information.

Data

Policy information about availability of data

All manuscripts must include a data availability statement. This statement should provide the following information, where applicable:

- Accession codes, unique identifiers, or web links for publicly available datasets
- A description of any restrictions on data availability
- For clinical datasets or third party data, please ensure that the statement adheres to our policy

The data for this manuscript's analyses were compiled by the authors from the studies identified in the systematic review. You can access the data on the Open Science Framework repository at https://osf.io/jkd4v/

Searches were conducted on the following databases:
PsycINFO (https://www.apa.org/pubs/databases/psycinfo)
Medine (https://www.nlm.nih.gov/medline/medline_home.html)
Embase (https://www.elsevier.com/en-gb/products/embase)
Psychology and Behavioral Science Collection (https://www.ebsco.com/products/research-databases/psychology-behavioral-sciences-collection)
Web of Science (https://clarivate.com/academia-government/scientific-and-academic-research/research-discovery-and-referencing/web-of-science/)
TRIP (https://www.tripdatabase.com/)

Research involving human participants, their data, or biological material

Policy information about studies with human participants or human data. See also policy information about sex, gender (identity/presentation), and sexual orientation and race, ethnicity and racism.

Reporting on sex and gender	N/A
Reporting on race, ethnicity, or other socially relevant groupings	N/A
Population characteristics	N/A
Recruitment	N/A
Ethics oversight	N/A

Note that full information on the approval of the study protocol must also be provided in the manuscript.

Field-specific reporting

Please select the one below that is the best fit for your research. If you are not sure, read the appropriate sections before making your selection.

☐ Life sciences ☒ Behavioural & social sciences ☐ Ecological, evolutionary & environmental sciences

For a reference copy of the document with all sections, see nature.com/documents/nr-reporting-summary-flat.pdf

Behavioural & social sciences study design

All studies must disclose on these points even when the disclosure is negative.

Study description

Systematic review and quantitative meta-analysis

Research sample

89 randomised controlled trials using social norms messaging in developed countries to change health behaviours among 16+ year-olds. Of the 89 studies included, 22 focused on diet, 12 on screening, 10 on vaccination, 10 on alcohol consumption, 10 on prescribing, nine on physical activity, and four on sexual health. There were 12 studies that we grouped as “other”; three studies on hand hygiene, three on sunscreen use, two on organ donation, two on appointment attendance, one on mental health, and one on smoking. The three studies on sunscreen use were part of the same paper.

Most studies (n = 47) reported on interventions in the general population. There were 23 studies that focused specifically on college students, 13 on healthcare professionals, and six on clinical patients. The studies included diverse delivery methods for social norms interventions: 29 used physical materials (letters or printed leaflets), 33 were delivered on-screen through mobile apps, websites, or similar displays, 10 were sent via email or text messages, and 10 employed multiple modalities or audio, such as spoken word combined with images or text. Additionally, seven interventions delivered social norms messages through posters or signs.

Interventions included various types of both descriptive and injunctive social norms messages. The majority of studies (n = 79) concentrated on descriptive social norms messaging, while four studies utilised injunctive social norms messaging (e.g. “A lot of people aren’t aware that the typical student thinks their peers should eat five servings of fruits and vegetables each day. Students think you should eat more fruit and vegetables than you’d expect.”9), and six studies employed a combination of both descriptive and injunctive social norms messages (e.g. “You thought that _____ % of college students try to avoid consuming sugar sweetened drinks. On average actually 90% of college students try to avoid consuming sugar-sweetened drinks.”21).

Sampling strategy

Randomised controlled trials using social norms messaging in developed countries to change health behaviours among 16+ year-olds.
Inclusion Criteria
Study type: Randomised controlled trial (RCT)
Language: Reported in English
Population: People aged 16 or older in developed countries (see the United Nations Conference on Trade and Development 2023 country classifications for further details)
Intervention:
Social norms message interventions targeting single health behaviours alone or multiple health behaviours, or healthcare workers’

	behaviours.<br>Includes messages that provide normative information about the behaviour of others within a relevant reference group, which may include social comparisons (target performance relative to a reference group), social proof (information about what the reference group does), injunctive norms (information about what ought to be done) or a combination thereof.<br>Messages can be delivered through any modality; and they can include pictures, as well as text or verbal messages.<br>It must be possible to assess the effectiveness of the social norms aspect of the intervention, so if there is a multi-component intervention, then the control condition must allow the effectiveness of the social norm component to be assessed, or the social norms element must be the main active ingredient of the intervention. All types of controls were considered, including studies with both active and passive controls.<br>Outcomes: The study should include objective behavioural outcomes or intention to perform behavioural outcomes. Examples of relevant outcomes: alcohol consumption, exercise or physical activity, fruit and vegetable consumption, prescribing, handwashing, uptake of screening, uptake of health checks, and uptake of vaccination.
Data collection	A combination of focussed searches were undertaken, including searches of bibliographic databases, and searches for grey literature.<br>Searches were conducted on the following databases:<br>PsycINFO<br>Medline<br>Embase<br>Psychology and Behavioral Science Collection<br>Web of Science<br>TRIP<br>Cochrane<br>We also searched for grey literature, by asking for studies from providers on the Cabinet Office Behavioural Insights procurement framework, and behavioural science research and consultancy groups, including the Behavioural Insights Team (BIT), Penn SoNG (University of Pennsylvania), CogCo (the Cognition Company Ltd) and Behaviour Change for Good (Wharton), and by searching PROSPERO for other registered reviews that might be relevant and asking their authors for reference lists.<br><br>Search terms and limits<br>Search terms were derived from the inclusion criteria in relation to study design (i.e. RCT), population (adults), intervention (social norms) and outcomes (health behaviours). We used both free text and thesaural terms. They were combined using Boolean and proximity operators to search the electronic databases stated above62 as follows (also see Supplementary Information):<br>[social norms OR ((social OR descriptive OR injunctive OR subjective) AND (norm OR norms)) OR ((peer OR family OR social) AND influence*]<br>AND<br>[letter OR text messaging OR (feedback OR leaflet* OR campaign* OR program* or letter* OR text* OR email OR e-mail OR (change* behavio?r*))]<br>AND<br>[(drug prescriptions OR prescrib* OR antimicrobial stewardship OR ((antimicrobial OR anti-microbial) stewardship))<br>OR<br>(infection control* OR (hand* AND (wash* OR hygiene))<br>OR<br>(overweight OR obesity OR (overweight OR obes*) OR (weight AND (control OR gain OR loss))<br>OR<br>(exercise OR sedentary behavio?r OR (physical activity OR exercis* OR “resistance training”)<br>OR<br>(diet OR feedback behaviour OR (snack* OR diet* OR nutrition) OR “eating behavio?r” OR ((fruit OR vegetable*) AND (consum* OR eat*))<br>OR<br>(sexual behaviour OR unsafe sex) OR (sexual AND (health OR behavio?r)) OR (“condom use” OR “using condom*” OR “safe sex”)<br>OR<br>(oral health OR ((oral OR dental) AND (health OR hygiene OR care)))<br>OR<br>(substance-related disorders OR (“drug use” OR “using drugs” OR “drug abuse” OR “drug misuse” OR “drug mis-use”) OR (drug* AND tak*))<br>OR<br>((alcoholic intoxication OR alcoholism OR binge drinking) OR (“binge drinking” OR “alcohol consum*” OR “alcohol misuse” OR “alcohol mis-use”))<br>OR<br>(screen time OR (screen viewing OR “screen time”))<br>OR<br>(public health OR “public health”)]<br>AND<br>[RCT OR “randomi?ed controlled trial*”]<br><br>Several limits were placed on the search, including a language filter to select only studies published in English, and an RCT filter to select only RCTs, since we anticipated a large number of studies and wanted to restrict to the gold standard for provision of evidence of effectiveness.<br><br>Exploratory searches were conducted in February 2021 and primary searches were conducted in March 2021 and re-run in April 2022 to guarantee that newly published studies are included in the systematic review. Our cut off date for retrieval of papers and grey literature for the first search was 31st May 2021 and 17th June 2022 for the updated search. A final database search was conducted in March 2024.
Timing	Exploratory searches were conducted in February 2021 and primary searches were conducted in March 2021 and re-run in April 2022 to guarantee that newly published studies are included in the systematic review. Our cut off date for retrieval of papers and

	grey literature for the first search was 31st May 2021 and 17th June 2022 for the updated search. A final database search was conducted in March 2024.
Data exclusions	Studies were excluded if:<br>They were conference abstracts, unpublished theses, discussion papers, editorials, policy articles, and epidemiological, cross-sectional or longitudinal observational studies or non-randomised controlled trials.<br>They did not include a social norms approach to health behaviour change.<br>Social norms interventions did not target a health behaviour.<br>It was not possible to isolate the effect of social norms, e.g., a multi-component intervention with a no-intervention control where the social norms component was not the main active ingredient.<br>They targeted the following populations or health behaviours: defecation, Female genital mutilation (FGM), alcohol or cannabis consumption in college students or school students, populations in developing countries.<br>If the population whose behaviour was targeted included adolescents or children under the age of 16.
Non-participation	No participants were involved in the study.
Randomization	This is a meta-analysis so randomisation was not applicable as we did not use any experimental methods.

Reporting for specific materials, systems and methods

We require information from authors about some types of materials, experimental systems and methods used in many studies. Here, indicate whether each material, system or method listed is relevant to your study. If you are not sure if a list item applies to your research, read the appropriate section before selecting a response.

Materials & experimental systems

n/a	Involved in the study	
☒	☐	Antibodies
☒	☐	Eukaryotic cell lines
☒	☐	Palaeontology and archaeology
☒	☐	Animals and other organisms
☒	☐	Clinical data
☒	☐	Dual use research of concern
☒	☐	Plants

Methods

n/a	Involved in the study	
☒	☐	ChIP-seq
☒	☐	Flow cytometry
☒	☐	MRI-based neuroimaging

Plants

Seed stocks	*Report on the source of all seed stocks or other plant material used. If applicable, state the seed stock centre and catalogue number. If plant specimens were collected from the field, describe the collection location, date and sampling procedures.*
Novel plant genotypes	*Describe the methods by which all novel plant genotypes were produced. This includes those generated by transgenic approaches, gene editing, chemical/radiation-based mutagenesis and hybridization. For transgenic lines, describe the transformation method, the number of independent lines analyzed and the generation upon which experiments were performed. For gene-edited lines, describe the editor used, the endogenous sequence targeted for editing, the targeting guide RNA sequence (if applicable) and how the editor was applied.*
Authentication	*Describe any authentication procedures for each seed stock used or novel genotype generated. Describe any experiments used to assess the effect of a mutation and, where applicable, how potential secondary effects (e.g. second site T-DNA insertions, mosiacism, off-target gene editing) were examined.*
